# Extracellular Vesicle-Mediated Delivery of Mitochondrial Circular RNA MTCO2 Protects against Cerebral Ischemia by Modulating mPTP-Dependent Ferroptosis

**DOI:** 10.34133/research.1232

**Published:** 2026-04-14

**Authors:** Jialei Yang, Shipo Wu, Miao He

**Affiliations:** ^1^ Department of Neurology, China National Clinical Research Center for Neurological Diseases, Beijing Tiantan Hospital, Capital Medical University, Beijing, China.; ^2^ National Key Laboratory of Advanced Biotechnology, Academy of Military Medical Sciences, Beijing, China.

## Abstract

Ischemic stroke remains a major cause of mortality and long-term disability, with few effective neuroprotective treatments currently available. Ferroptosis, an iron-dependent form of regulated cell death marked by lipid peroxidation, is increasingly recognized as a driver of neuronal damage. However, the mitochondrial mechanisms linking ischemia to ferroptosis remain poorly defined. Here, we identify circMTCO2, a mitochondria-encoded circular RNA (circRNA), as a novel endogenous modulator of neuronal ferroptosis. circMTCO2 expression is dynamically down-regulated following cerebral ischemia/reperfusion in vitro and in vivo. Mechanistically, circMTCO2 interacts with adenine nucleotide translocase 1 (ANT1), a key regulator associated with the mitochondrial permeability transition pore (mPTP), thereby inhibiting mPTP opening and suppressing mitochondrial reactive oxygen species release. Disruption of the binding site abolishes circMTCO2–ANT1 interaction and eliminates the protective effects of circMTCO2. To restore and enhance this intrinsic defense mechanism, we developed a dual-targeting extracellular vesicle system (RVG-EV^mt-RNA^) capable of delivering circMTCO2 specifically to neuronal mitochondria. Systemic administration of RVG-EV^mt-RNA^ decreased infarct volume, attenuated ferroptosis-associated injury, and improved neurological function in a mouse model of ischemic stroke, without inducing systemic toxicity. These findings establish circMTCO2 as a previously unrecognized mitochondrial circRNA that regulates ferroptosis by modulating mPTP activity and provide a proof of concept that organ-to-organelle circRNA delivery can be leveraged as a precision neuroprotective strategy for ischemic stroke.

## Introduction

Ischemic stroke is a leading cause of death and long-term disability worldwide, driven by a complex cascade of pathological events following the sudden interruption of cerebral blood flow (CBF) [1]. While reperfusion therapies such as thrombolysis and thrombectomy offer benefits when delivered within a narrow time window, they are applicable to only a limited subset of patients. Critically, there are no effective neuroprotective treatments available to limit neuronal death or enhance recovery [2]. Addressing this unmet need requires a deeper understanding of the molecular and organellar mechanisms that govern ischemic neuronal death.

Ferroptosis, a distinct form of regulated cell death first defined in 2012 [3], has emerged as a key contributor to neuronal injury in various neurological disorders, including ischemic stroke [4], intracerebral hemorrhage [5,6], and neurodegenerative diseases such as Parkinson’s disease [7] and amyotrophic lateral sclerosis [8]. Ferroptosis is characterized by iron-dependent lipid peroxidation and is driven by metabolic dysfunction, redox imbalance, and mitochondrial perturbation [9]. In neurons, ferroptosis is fueled by intracellular iron accumulation and excessive reactive oxygen species (ROS), particularly mitochondrial ROS (mtROS), which initiate widespread oxidative damage. Depletion of the antioxidant enzyme and the disruption of glutathione (GSH) homeostasis further exacerbate lipid peroxidation, ultimately compromising membrane integrity and triggering cell death. Although ferroptosis contributes to neuronal demise at multiple stages of ischemia [10], whether intrinsic mechanisms exist to suppress ferroptosis and preserve neuronal viability remains largely unknown. Uncovering these endogenous protective pathways and enhancing them therapeutically could open new avenues for intervention in ischemic stroke and related neurological disorders.

Mitochondria play a central and multifaceted role in ferroptosis, acting as both a major source and a key target of oxidative stress [11]. Under physiological conditions, the mitochondrial electron transport chain supports energy production while generating modest levels of ROS that are efficiently neutralized by endogenous antioxidant systems [12,13]. During ischemia/reperfusion, however, mitochondrial dysfunction drives excessive mtROS generation and can trigger the opening of the mitochondrial permeability transition pore (mPTP) [14,15]. The mPTP is functionally defined as a regulated, high-conductance permeability pathway of the inner mitochondrial membrane. Current evidence supports a multiprotein model in which cyclophilin D functions as a key regulatory factor; inner-membrane components such as adenine nucleotide translocase (including adenine nucleotide translocase 1 [ANT1]) are closely linked to pore gating, and outer-membrane proteins (such as voltage-dependent anion channels [VDACs]) have also been proposed to participate in a context-dependent manner [14,16]. Sustained mPTP opening promotes mtROS to escape into the cytosol and facilitates the propagation of oxidative stress signals, thereby accelerating lipid peroxidation and ferroptotic injury [17]. While several mitochondrial enzymes, including glutathione peroxidase 4, dihydroorotate dehydrogenase, and superoxide dismutase 2, have been implicated in buffering mitochondrial oxidative stress [18], the endogenous upstream mechanisms that tune mPTP opening and mtROS propagation under ischemic stress remain incompletely understood.

Adding further complexity, mitochondria harbor their own genome and transcriptional machinery, and recent findings suggest that they can generate mitochondrial circular RNAs (mt-circRNAs), which are covalently closed noncoding RNAs with high stability and regulatory capacity [19,20]. In the brain, circular RNAs (circRNAs) exhibit spatial and temporal expression patterns and have been implicated in neuronal development, synaptic plasticity, and neuroprotection [21]. While nuclear-derived circRNAs have been shown to regulate inflammation, apoptosis, and blood–brain barrier integrity in stroke models [22], the roles of mitochondria-derived circRNAs remain largely unexplored. Their unique localization and origin suggest that they may be strategically positioned to influence mitochondrial integrity, redox signaling, and ferroptotic susceptibility.

Here, we identify circMTCO2, a mitochondria-encoded circRNA, as an endogenous neuroprotective factor that limits neuronal ferroptosis during cerebral ischemia/reperfusion. We show that circMTCO2 directly binds to ANT1, thereby restricting pore opening and curbing mtROS accumulation. Importantly, ischemic injury dynamically down-regulates circMTCO2, weakening this intrinsic defense. To restore and amplify this protective mechanism, we developed a dual-targeting extracellular vesicle (EV) system capable of delivering circMTCO2 to neuronal mitochondria. This work provides a proof of concept for harnessing and enhancing mitochondrial-RNA-based defenses, revealing mt-circRNAs as both mechanistic regulators and therapeutic targets for precision neuroprotection in stroke.

## Results

### Ischemic stroke triggers neuronal lipid peroxidation and ferroptosis

**Fig. 1.**
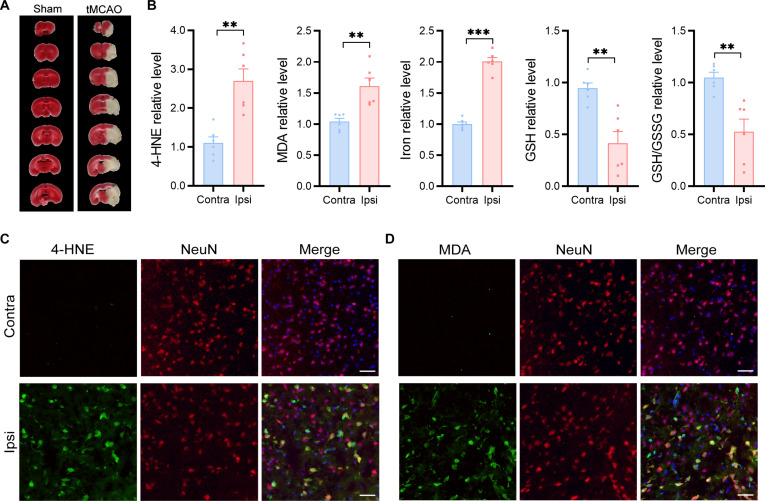
Ischemia/reperfusion injury induces neuronal lipid peroxidation and ferroptosis in the mouse brain. (A) Representative triphenyl tetrazolium chloride (TTC) staining of brain sections at 24 h postinjury (hpi). The white region indicates infarcted tissue. (B) Quantification of 4-hydroxynonenal (4-HNE), malondialdehyde (MDA), total iron, glutathione (GSH), and the GSH/glutathione disulfide (GSSG) ratio in the ipsilateral (Ipsi) and contralateral (Contra) hemispheres at 24 hpi. (C and D) Immunofluorescence staining of 4-HNE/NeuN and MDA/NeuN in the ipsilateral contralateral hemispheres at 24 hpi. Scale bars, 30 μm. Data are presented as mean ± standard error of the mean (SEM) (*n* = 6 per group). Statistical analysis was performed using an unpaired 2-tailed Student *t* test. **P < 0.01; ***P < 0.001.

To investigate the role of neuronal ferroptosis in cerebral ischemia/reperfusion injury, we established a mouse transient middle cerebral artery occlusion (tMCAO) model and confirmed infarction by triphenyl tetrazolium chloride (TTC) staining at 24 h postinjury. Representative images are shown in Fig. 1A, with the white region indicating infarcted tissue. Ferroptosis-associated markers, including 4-hydroxynonenal (4-HNE), malondialdehyde (MDA), total iron, GSH, and GSH/glutathione disulfide (GSSG) ratio, were assessed in sham animals as well as in the ipsilateral (Ipsi) and contralateral (Contra) hemispheres of tMCAO mice. No significant differences were observed between the sham group and the contralateral hemisphere of tMCAO mice (Fig. S1A), indicating minimal oxidative damage in nonischemic tissue. In contrast, compared to the contralateral side, the ipsilateral hemisphere exhibited significantly elevated levels of 4-HNE, MDA, and total iron, along with decreased GSH levels and a reduced GSH/GSSG ratio (Fig. 1B). Immunofluorescence staining for NeuN, 4-HNE, and MDA further demonstrated colocalization of lipid peroxidation products with neurons in the ischemic cortex (Fig. 1C and D, lower panels). In contrast, no significant staining of 4-HNE and MDA was observed in the contralateral cortex (Fig. 1C and D, upper panels) and the cortex of sham animals (Fig. S1B), supporting that cerebral ischemia/reperfusion induces neuronal lipid peroxidation and ferroptosis-associated injury.

### OGD-induced mtROS promote ferroptosis-associated injury through mPTP dysfunction

**Fig. 2.**
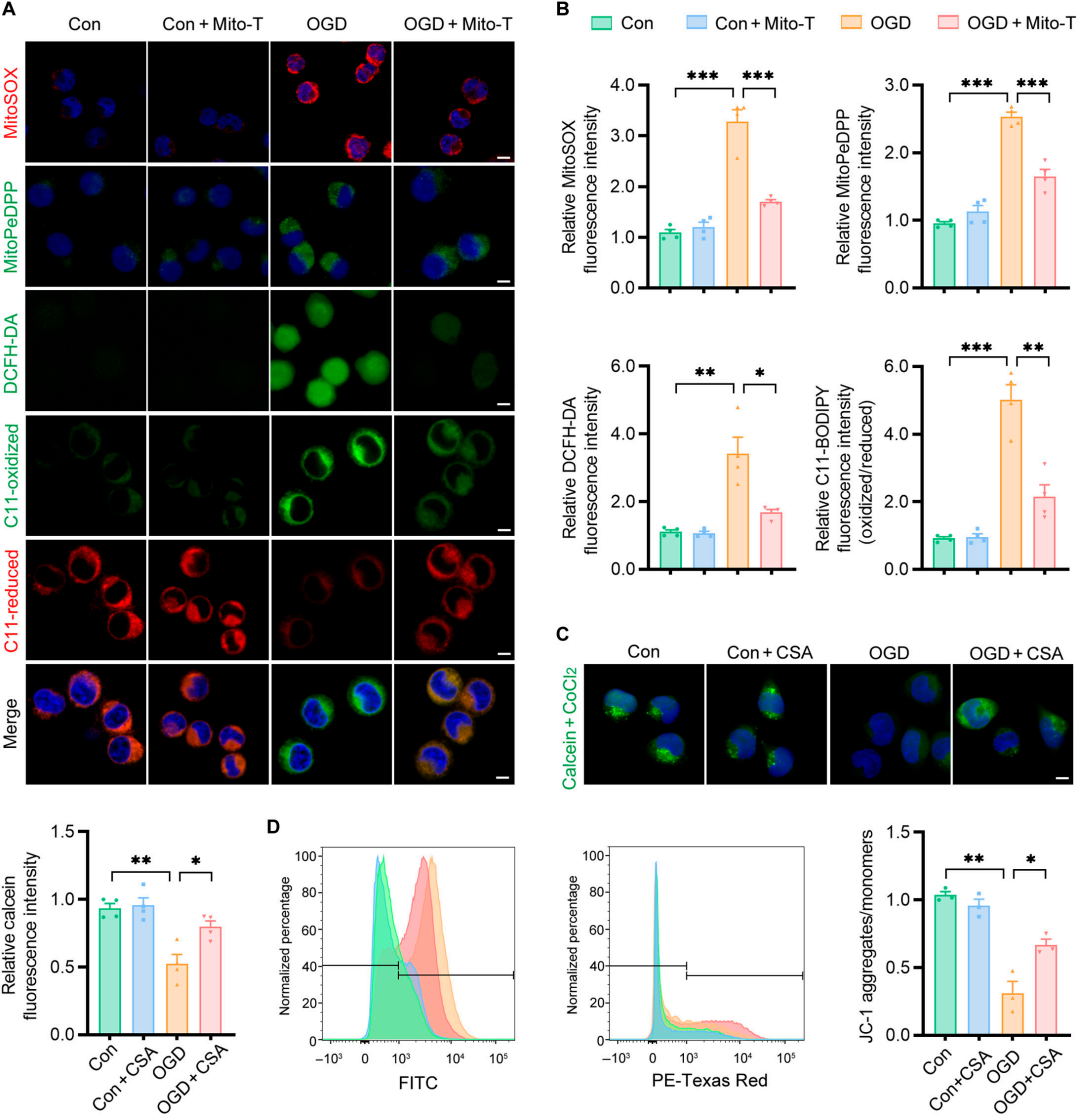
Mitochondrial reactive oxygen species (ROS) and mitochondrial permeability transition pore (mPTP) opening promote ferroptosis in oxygen-glucose deprivation (OGD)-treated neurons. (A) Representative fluorescence images of MitoSOX, MitoPeDPP, 2′,7′-dichlorofluorescein diacetate (DCFH-DA), and C11-BODIPY (oxidized and reduced forms) in the control (Con) and OGD groups, with or without Mito-TEMPO (Mito-T). (B) Quantification of fluorescence intensity for the indicated oxidative stress markers in each condition. (C) Representative fluorescence images and quantification of calcein fluorescence in the control and OGD groups with or without cyclosporin A (CSA), using the calcein–CoCl_2_ mPTP quenching assay to assess mPTP opening. (D) Representative flow cytometry histograms and quantification of JC-1 staining to assess mitochondrial membrane potential (ΔΨm). Scale bars, 5 μm. Data are presented as mean ± standard error of the mean (SEM) (*n* = 3 to 4 per group). Statistical analysis was performed using an unpaired 2-tailed Student *t* test. *P < 0.05; **P < 0.01; ***P < 0.001.

Mitochondria are a primary source of ROS, and impaired mitochondrial function leads to excessive mtROS production, a known trigger of ferroptosis. To explore the role of mtROS in neuronal ferroptosis, we established an oxygen-glucose deprivation (OGD) model followed by reoxygenation in Neuro2a (N2a) cells. Fluorescent probes revealed a marked increase in mtROS (MitoSOX) and mitochondrial lipid peroxidation (MitoPeDPP) in OGD-treated cells (Fig. 2A and B). Additionally, the levels of cytosolic ROS (cROS; measured by 2′,7′-dichlorofluorescein diacetate [DCFH-DA]) and the oxidized/reduced fluorescence ratio of C11-BODIPY also increased, confirming elevated oxidative stress and lipid peroxidation. Treatment with Mito-TEMPO (Mito-T), an mtROS inhibitor, significantly reduced mtROS, cROS, and lipid peroxidation, supporting an important role for mtROS in OGD-induced ferroptosis.

mPTP opening is a key regulator of mitochondrial function and mtROS production [16,17]. Using the calcein–CoCl_2_ quenching assay, we demonstrated that cyclosporin A (CSA), an mPTP inhibitor, effectively blocked mPTP opening (Fig. 2C). CSA also reduced mtROS, mitochondrial lipid peroxidation, cROS, and C11-BODIPY oxidation while restoring mitochondrial membrane potential (ΔΨm), as assessed by JC-1 staining (Fig. 2D and Fig. S2A and B). Furthermore, both Mito-T and CSA treatment significantly improved cell viability under OGD conditions, as measured by the Cell Counting Kit-8 (CCK-8) assay and Live/Dead cell staining (Fig. S2C to E). Collectively, these findings demonstrate that mtROS production and mPTP dysfunction are closely associated with ferroptotic injury under OGD conditions and that their inhibition mitigates neuronal oxidative damage.

### circMTCO2 is a stable mt-circRNA down-regulated by ischemic stroke

**Fig. 3.**
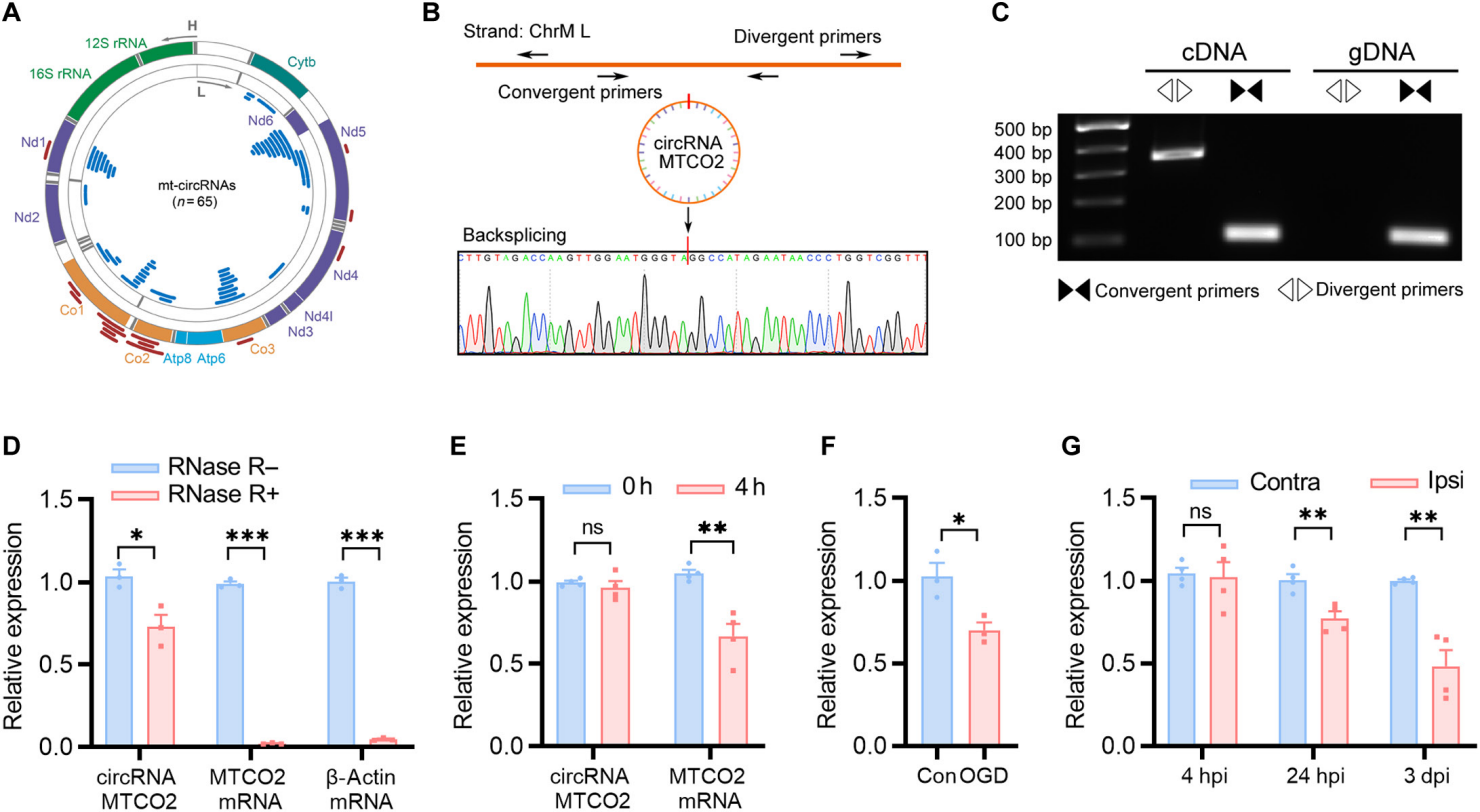
circMTCO2 is a stable mitochondrial circular RNA (mt-circRNA) down-regulated by ischemic stroke. (A) Circular representation of the mouse mitochondrial genome showing the distribution of 65 mt-circRNAs. The outer and inner rings correspond to the mitochondrial heavy (H) and light (L) strands, respectively. Red arcs represent mt-circRNAs originating from the H strand, and blue arcs represent those from the L strand. (B) Schematic of divergent primer design targeting the backsplice junction (BSJ) of circMTCO2 and representative Sanger sequencing results. (C) Validation of circMTCO2 in mouse brain using polymerase chain reaction (PCR). Divergent primers spanning the BSJ amplify circMTCO2 only in complementary DNA (cDNA), not in genomic DNA (gDNA), confirming its circular structure. Convergent primers amplify linear MTCO2 in both cDNA and gDNA as a control. (D) Quantitative reverse transcription polymerase chain reaction (qRT-PCR) analysis showing that circMTCO2 is relatively resistant to RNase R digestion, whereas linear MTCO2 messenger RNA (mRNA) and β-actin mRNA (control transcripts) are degraded, supporting the circularity and stability of circMTCO2. (E) Time-course qRT-PCR showing greater stability of circMTCO2 than that of MTCO2 mRNA following actinomycin D (Act D) treatment in Neuro2a (N2a) cells. (F) Relative expression of circMTCO2 in control and oxygen-glucose deprivation (OGD)-treated N2a cells. (G) qRT-PCR analysis of circMTCO2 expression in ipsilateral (Ipsi) and contralateral (Contra) hemispheres at 4 h, 24 h, and 3 d postinjury. Data are presented as mean ± standard error of the mean (SEM) (*n* = 3 to 4 per group). Statistical analysis was performed using an unpaired 2-tailed Student *t* test. *P < 0.05; **P < 0.01; ***P < 0.001; ns, not significant.

Recent studies have highlighted the significant roles of mt-circRNAs in regulating mitochondrial function, and their dysregulation has been implicated in various pathophysiological conditions [19,23,24]. Given the high abundance of mitochondria in the brain, we performed mt-circRNA sequencing in mouse brain tissue and identified 65 mt-circRNAs, which were distributed across both the heavy and light strands of the mitochondrial genome (Fig. 3A). Among these, circRNAs derived from the mitochondrial gene *MTCO2* have been shown to influence mtROS production in human liver fibroblasts and to be associated with lymphocytic leukemia [25,26]. To confirm its presence in the brain, we designed divergent and convergent primers targeting the backsplice junction of circMTCO2. Sanger sequencing of the polymerase chain reaction (PCR) product confirmed the existence of circMTCO2 (Fig. 3B). This was further validated by PCR analysis of complementary DNA (cDNA) and genomic DNA (gDNA) from mouse brain, as divergent primers amplified a product in cDNA, but not in gDNA, confirming the circular nature of circMTCO2 (Fig. 3C). To assess the stability of circMTCO2, we treated total RNA with RNase R. As expected, circMTCO2 was relatively resistant to RNase R treatment, whereas linear MTCO2 messenger RNA (mRNA) and β-actin mRNA were nearly completely degraded (Fig. 3D). Similarly, 4 h of transcriptional inhibition with actinomycin D (Act D) confirmed that circMTCO2 is more stable than its linear counterpart in N2a cells (Fig. 3E). To determine its relevance to ischemia, we measured circMTCO2 levels by quantitative reverse transcription polymerase chain reaction (qRT-PCR) in OGD-treated N2a cells and observed a significant reduction (Fig. 3F). In vivo, circMTCO2 expression in the ipsilateral hemisphere of tMCAO mice was significantly decreased at 24 h postinjury and remained reduced through day 3 (Fig. 3G). Taken together, these findings identify circMTCO2 as a stable, mitochondria-derived circRNA whose expression is dynamically regulated. Importantly, the significant down-regulation of circMTCO2 in both in vitro and in vivo models of ischemic stroke suggests a potential role in the pathophysiology of ischemic brain injury.

### Engineering an EV platform for neuron-to-mitochondria targeted delivery of circMTCO2

To investigate the functional role of circMTCO2 in neuronal ferroptosis and ischemic stroke, we developed a dual-targeting EV delivery system capable of transporting circMTCO2 into neuronal mitochondria. This approach utilized the rabies virus glycoprotein (RVG) peptide, which specifically binds to acetylcholine receptors, facilitating selective targeting of EVs to neuronal cells. The RVG peptide was fused to the EV membrane protein lysosome-associated membrane glycoprotein 2b (Lamp2b). Lentiviruses expressing RVG–Lamp2b and circMTCO2 with or without green fluorescent protein (GFP) were cotransduced into suspension 293F cells, followed by puromycin and hygromycin B selection to establish a stable cell line. EVs were isolated from the culture supernatant by sequential ultracentrifugation and filtered through a 0.22-μm filter to ensure sterility. EV^Ctrl^ refers to EVs harvested from the supernatant of untransduced 293F cells, serving as the control group in our experiments.

**Fig. 4.**
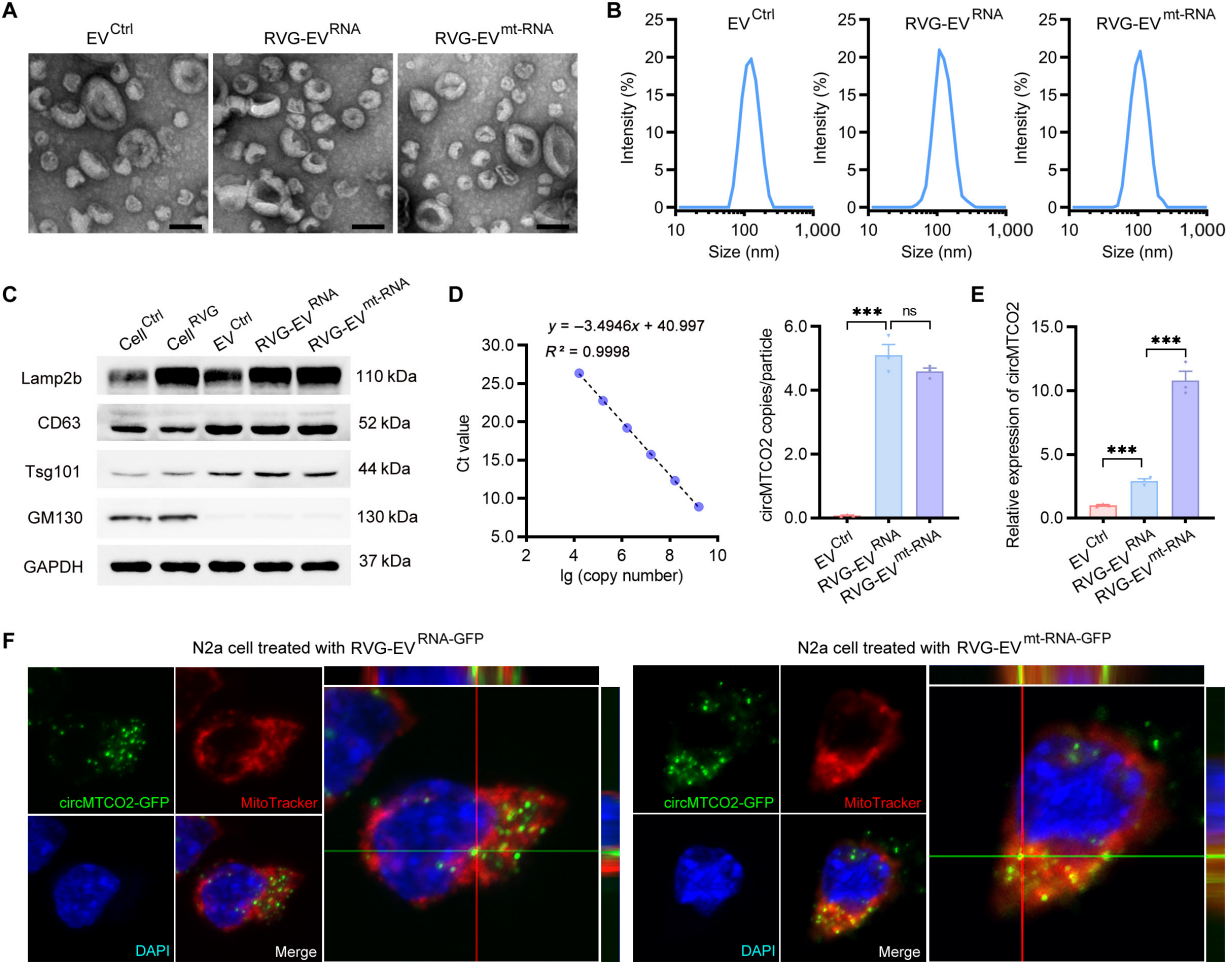
The dual-targeting extracellular vesicle (EV) platform enables specific delivery of circMTCO2 to neuronal mitochondria. (A) Transmission electron microscopy images of EV^Ctrl^, RVG-EV^RNA^, and RVG-EV^mt-RNA^. Scale bars, 100 nm. (B) Nanoparticle size distribution of the indicated EVs. (C) Western blot of EV markers (lysosome-associated membrane glycoprotein 2b [Lamp2b], CD63, and tumor susceptibility gene 101 protein [Tsg101]) and the Golgi marker 130 kDa cis-Golgi matrix protein 1 (GM130) in parental cells and purified EVs. (D) Absolute quantification of circMTCO2 copies per EV by quantitative polymerase chain reaction (qPCR). (E) Relative expression of circMTCO2 in mitochondrial fractions of Neuro2a (N2a) cells treated with the indicated EVs for 24 h. (F) Representative confocal fluorescence microscopy images of N2a cells treated with RVG-EV^RNA-GFP^ or RVG-EV^mt-RNA-GFP^. Mitochondria were stained with MitoTracker, and nuclei were counterstained with 4′,6-diamidino-2-phenylindole (DAPI). Notable colocalization was observed in the RVG-EV^mt-RNA-GFP^ group. Data are presented as mean ± standard error of the mean (SEM) (*n* = 3 per group). Statistical analysis was performed using an unpaired 2-tailed Student *t* test. ***P < 0.001; ns, not significant.

To further direct circMTCO2 to mitochondria, we employed triphenylphosphonium (TPP)–poly-d-lysine (PDL) conjugates, which are known to exhibit low cytotoxicity and high affinity for RNA [25,27]. TPP–PDL was electroporated into RVG-EV^RNA^ to generate the mitochondria-targeted RVG-EV^mt-RNA^. Transmission electron microscopy confirmed that EV morphology was unchanged by these modifications (Fig. 4A). Nanoparticle size distribution analysis showed similar diameters across EV groups: 115.1 ± 1.3 nm (EV^Ctrl^), 120.1 ± 2.1 nm (RVG-EV^RNA^), and 112.9 ± 1.8 nm (RVG-EV^mt-RNA^) (Fig. 4B). Western blotting for classical EV markers (Lamp2b, CD63, and tumor susceptibility gene 101 protein [Tsg101]) and the Golgi marker 130 kDa cis-Golgi matrix protein 1 (GM130) validated EV identity and purity (Fig. 4C). Next, we measured the amount of circMTCO2 encapsulated in EVs by absolute quantitative polymerase chain reaction (qPCR). The copy number of circMTCO2 per EV particle was 5.10 ± 0.33 in RVG-EV^RNA^ and 4.59 ± 0.11 in RVG-EV^mt-RNA^ (Fig. 4D). To assess mitochondrial delivery, N2a cells were treated with EV^Ctrl^, RVG-EV^RNA^, or RVG-EV^mt-RNA^ for 24 h, after which mitochondrial RNA was extracted. qRT-PCR revealed that RVG-EV^mt-RNA^ efficiently delivered circMTCO2 into mitochondria (Fig. 4E). To further confirm circMTCO2 delivery and subcellular localization, we generated GFP-labeled circMTCO2 constructs to obtain RVG-EV^RNA-GFP^ and RVG-EV^mt-RNA-GFP^, both of which exhibited characteristics similar to those of their unlabeled counterparts (Fig. S3). In cells treated with RVG-EV^mt-RNA-GFP^, circMTCO2-GFP localized specifically to mitochondria (Fig. 4F, right panel), whereas in the absence of the TPP–PDL moiety, circMTCO2-GFP was more diffusely distributed in the cytoplasm (Fig. 4F, left panel). Together, these findings demonstrate that the sequential neuron-to-mitochondria targeting EV platform enables efficient and specific circMTCO2 delivery into neuronal mitochondria, providing a useful tool for investigating its role in oxidative injury and ferroptosis.

### Mitochondria-targeted circMTCO2 delivery attenuates neuronal ferroptosis following OGD

**Fig. 5.**
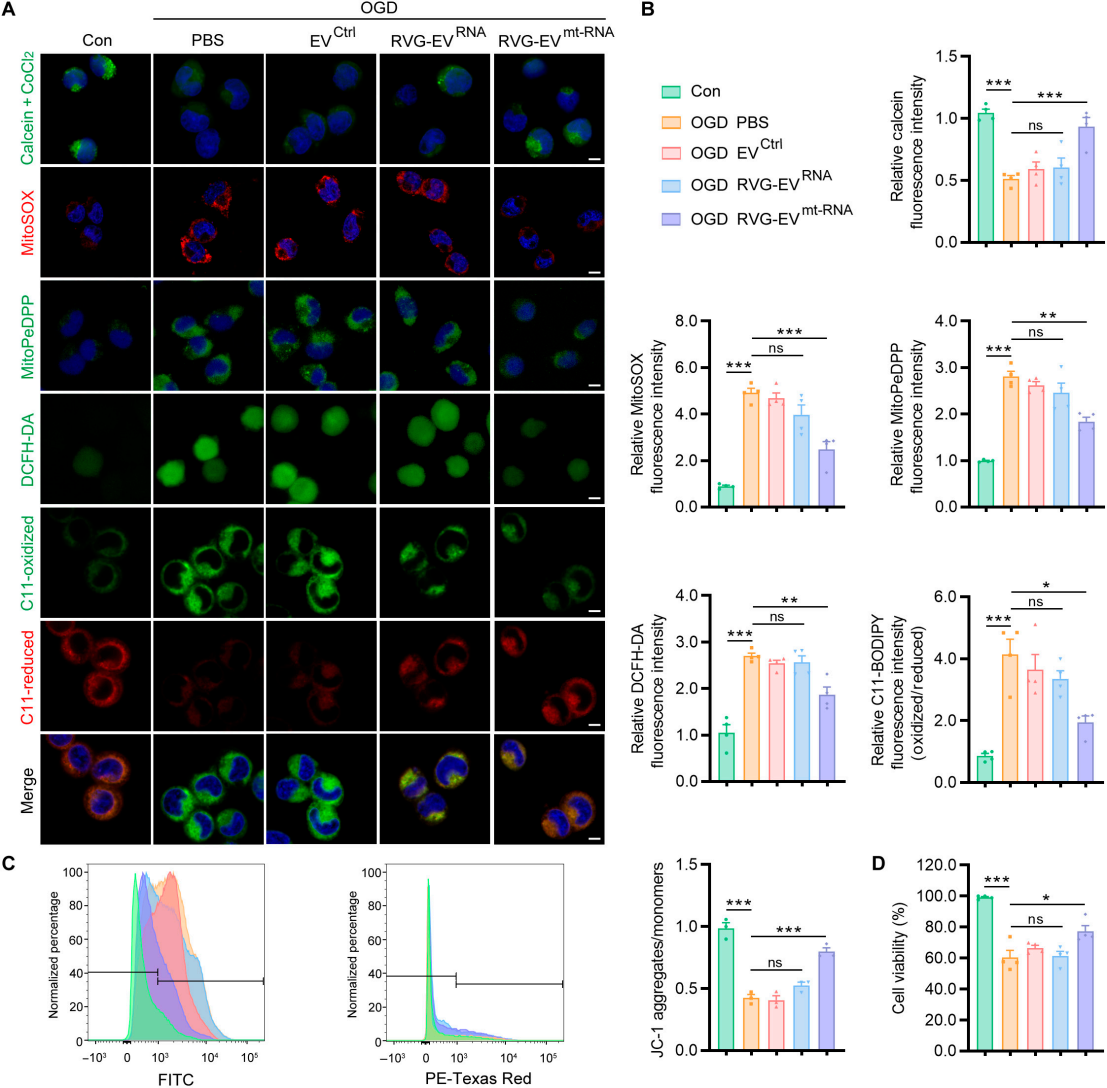
Mitochondria-targeted circMTCO2 delivery alleviates oxygen-glucose deprivation (OGD)-induced neuronal ferroptosis. (A) Representative fluorescence images of calcein, MitoSOX, MitoPeDPP, 2′,7′-dichlorofluorescein diacetate (DCFH-DA), and C11-BODIPY (oxidized and reduced forms) in Neuro2a (N2a) cells under control or OGD conditions, treated with phosphate-buffered saline (PBS), EV^Ctrl^, RVG-EV^RNA^, and RVG-EV^mt-RNA^. (B) Quantification of fluorescence intensities shown in panel (A). (C) Representative flow cytometry histograms and quantification of JC-1 staining to assess mitochondrial membrane potential (ΔΨm). (D) Cell viability assessed by the Cell Counting Kit-8 (CCK-8) assay. Scale bars, 5 μm. Data are presented as mean ± standard error of the mean (SEM) (*n* = 3 to 4 per group). Statistical analysis was performed using an unpaired 2-tailed Student *t* test and one-way analysis of variance (ANOVA) followed by Tukey’s post hoc test. *P < 0.05; **P < 0.01; ***P < 0.001; ns, not significant.

To investigate the effects of circMTCO2 on mitochondrial function and neuronal ferroptosis, we treated OGD-exposed N2a cells with EV^Ctrl^, RVG-EV^RNA^, or RVG-EV^mt-RNA^. Using the calcein–CoCl_2_ quenching assay, we found that treatment with RVG-EV^mt-RNA^ significantly restored calcein fluorescence intensity, indicating that circMTCO2 mitigates mPTP opening under OGD conditions (Fig. 5A). MitoSOX and MitoPeDPP staining showed that RVG-EV^mt-RNA^ reduced mtROS and mitochondrial lipid peroxidation. Similarly, DCFH-DA and oxidized/reduced C11-BODIPY probes demonstrated reduced cROS and cellular lipid peroxidation. JC-1 staining further confirmed that RVG-EV^mt-RNA^ partially restored mitochondrial membrane potential (ΔΨm), indicating improved mitochondrial integrity (Fig. 5A to C). Moreover, CCK-8 assays and Live/Dead cell staining demonstrated enhanced cell viability in RVG-EV^mt-RNA^-treated cells under OGD conditions, indicating that RVG-EV^mt-RNA^ effectively mitigates cell injury (Fig. 5D and Fig. S4A). To evaluate whether TPP–PDL itself exerts any intrinsic protective effects, we also included a control group treated with RVG-EV^mt^ (TPP–PDL-modified vesicles lacking circMTCO2). This group was tested across multiple assays under OGD conditions (Fig. S5). In all assays, RVG-EV^mt^ and RVG-EV^RNA^ produced comparable results, suggesting that TPP–PDL itself does not produce measurable mitochondrial or redox-related protection.

To further validate the protective effect of RVG-EV^mt-RNA^, we established an erastin-induced ferroptosis model. The results demonstrated that RVG-EV^mt-RNA^ significantly alleviated ferroptosis-associated injury, as evidenced by reduced oxidative stress and lipid peroxidation markers together with improved cell viability (Fig. S4B to D). These findings collectively demonstrate that mitochondria-targeted circMTCO2 delivery via RVG-EV^mt-RNA^ protects neurons from ferroptosis-associated injury by restoring mitochondrial function and redox balance, highlighting its therapeutic potential for ischemic stroke.

### circMTCO2 interacts with ANT1 to suppress mPTP opening under oxidative stress

**Fig. 6.**
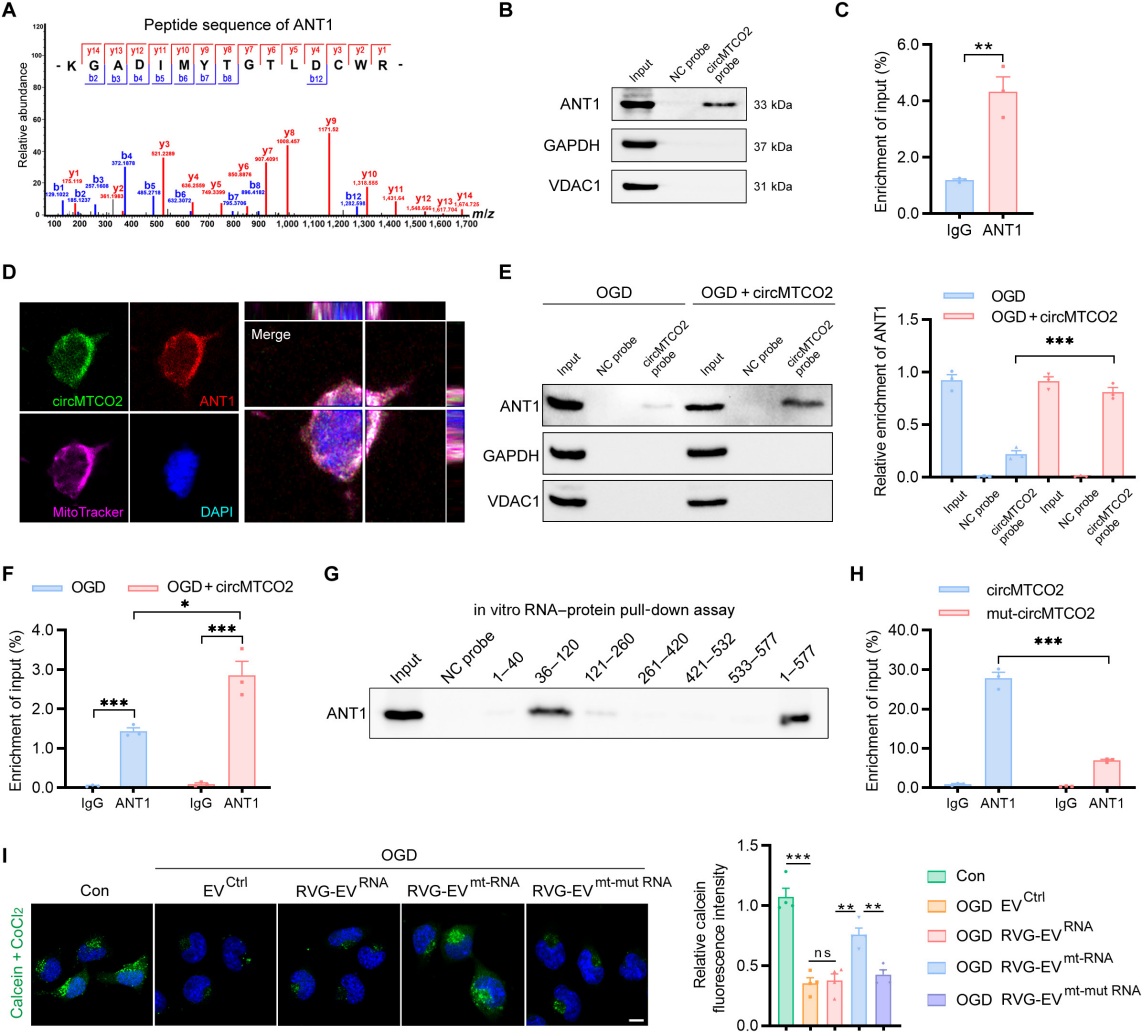
circMTCO2 interacts with adenine nucleotide translocase 1 (ANT1) to inhibit mitochondrial permeability transition pore (mPTP) opening and preserve mitochondrial integrity. (A) Liquid chromatography–tandem mass spectrometry (LC–MS/MS) identification of a representative peptide sequence of ANT1 pulled down by a biotinylated circMTCO2 probe in Neuro2a (N2a) cells. (B) Western blot validation of ANT1 pulled down by the biotinylated circMTCO2 probe, with glyceraldehyde-3-phosphate dehydrogenase (GAPDH) and voltage-dependent anion channel 1 (VDAC1) serving as cytoplasmic and mitochondrial controls, respectively. A biotin-labeled negative control (NC) probe was included to assess nonspecific binding. (C) RNA immunoprecipitation (RIP) using an antibody against ANT1, followed by quantitative reverse transcription polymerase chain reaction (qRT-PCR) for circMTCO2, showing enrichment of circMTCO2 in ANT1 immunoprecipitates. (D) Confocal fluorescence microscopy images showing colocalization of circMTCO2 detected by fluorescence in situ hybridization (FISH) with ANT1 and MitoTracker in the mitochondrial region. (E) Western blot and (F) RIP confirming enhanced ANT1–circMTCO2 association after circMTCO2 overexpression in oxygen-glucose deprivation (OGD) N2a cells. (G) In vitro RNA–protein pull-down using truncated circMTCO2 probes revealing the 36- to 120-nt region as essential for ANT1 binding. (H) RIP showing significantly decreased interaction between ANT1 and mutant circMTCO2 compared to full-length circMTCO2. (I) Calcein–CoCl_2_ quenching assay showing loss of mPTP-suppressive protection with mutant circMTCO2. Scale bar, 5 μm. Data are presented as mean ± standard error of the mean (SEM) (*n* = 3 to 4 per group). Statistical analysis was performed using an unpaired 2-tailed Student *t* test. *P < 0.05; **P < 0.01; ***P < 0.001; ns, not significant.

CircRNAs are emerging as key regulators of RNA-binding proteins and cellular processes. To elucidate the molecular mechanisms underlying the protective effects of circMTCO2, we conducted RNA pull-down assays in N2a cells using a biotinylated circMTCO2 probe, followed by liquid chromatography–tandem mass spectrometry (LC–MS/MS) analysis. ANT1, a key regulator of the mPTP, was identified as a potential binding partner of circMTCO2 (Fig. 6A). Western blotting confirmed the presence of ANT1 in the circMTCO2-associated protein complex (Fig. 6B). RNA immunoprecipitation (RIP) assays showed that circMTCO2 was specifically enriched in ANT1 immunoprecipitates, further supporting interaction between circMTCO2 and ANT1 (Fig. 6C). To investigate their subcellular localization, we performed circMTCO2 fluorescence in situ hybridization (FISH) combined with MitoTracker and ANT1 staining. Although submitochondrial resolution was not achieved, the results revealed substantial colocalization of circMTCO2 with ANT1 in the mitochondrial region (Fig. 6D).

Next, to test whether OGD impairs the interaction between endogenous circMTCO2 and ANT1, we performed RNA pull-down assays under OGD conditions. The results showed that OGD significantly reduces the binding between circMTCO2 and ANT1 (Fig. S6). To examine the regulatory dynamics of this interaction under oxidative stress, we overexpressed circMTCO2 in OGD-treated N2a cells. RNA pull-down and RIP assays demonstrated that circMTCO2 overexpression significantly enhanced the association between ANT1 and circMTCO2, restoring the interaction impaired by OGD (Fig. 6E and F). To identify the specific region of circMTCO2 involved in ANT1 binding, we predicted its secondary structure and interaction propensity using RNAfold and catRAPID (Fig. S7) and constructed a series of truncated circMTCO2 variants. In vitro RNA–protein pull-down assays identified the 36- to 120-nt region of circMTCO2 as essential for ANT1 binding (Fig. 6G). We then overexpressed full-length and mutant circMTCO2 in N2a cells. RIP assays showed that mutations disrupting the paired region of circMTCO2 markedly reduced the ANT1–circMTCO2 interaction (Fig. 6H). Finally, we assessed the functional significance of this interaction in regulating mPTP opening using the calcein–CoCl_2_ quenching assay. The results showed that the protective effect of RVG-EV carrying full-length circMTCO2 was significantly diminished when the binding-deficient mutant circMTCO2 was used (Fig. 6I). Together, these findings demonstrate that circMTCO2 binds ANT1 through a defined structural region and that this interaction is required for the suppression of mPTP opening under oxidative stress.

### Mitochondria-targeted circMTCO2 delivery reduces ischemic injury and promotes functional recovery

**Fig. 7.**
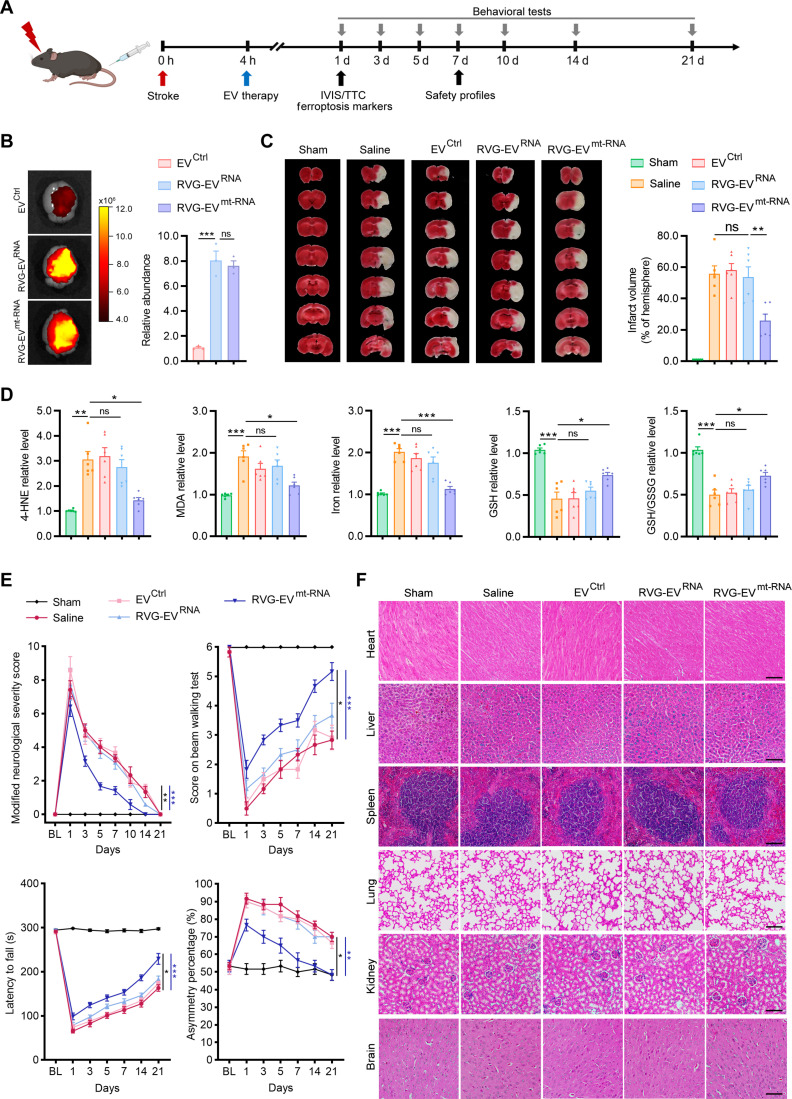
Mitochondria-targeted circMTCO2 delivery alleviates ischemic injury and improves functional recovery in mice. (A) Experimental timeline for stroke induction, therapeutic intervention, behavioral testing, and endpoint analyses. (B) Representative in vivo imaging system (IVIS) images and quantification of DiR (1,1ʹ-dioctadecyl-3,3,3ʹ,3ʹ-tetramethylindotricarbocyanine iodide)-labeled EV^Ctrl^, RVG-EV^RNA^, and RVG-EV^mt-RNA^ accumulation in the brain (*n* = 3 per group). (C) Representative triphenyl tetrazolium chloride (TTC) staining of brain sections and infarct volume quantification at 1 d postinjury (dpi) (*n* = 6 per group). (D) Quantification of oxidative stress and lipid-peroxidation-associated markers (4-hydroxynonenal [4-HNE], malondialdehyde [MDA], iron, glutathione [GSH], and the GSH/glutathione disulfide [GSSG] ratio) in ischemic hemispheres at 1 dpi (*n* = 6 per group). (E) Behavioral outcomes assessed by modified neurological severity score (mNSS) and beam walking, rotarod, and corner turn tests over 21 d (*n* = 6 per group). (F) Representative hematoxylin and eosin (H&E) staining of major organs (heart, liver, spleen, lung, kidney, and brain) at 7 dpi (*n* = 6 per group). Scale bars, 100 μm. Data are presented as mean ± standard error of the mean (SEM). Statistical analyses were performed using an unpaired 2-tailed Student *t* test and one-way analysis of variance (ANOVA) followed by Tukey’s post hoc test for panels (B) to (D). For panel (E), 2-way ANOVA (group × time) was used. Overall differences among the stroke groups (excluding sham) are indicated by black asterisks, and the saline versus RVG-EV^mt-RNA^ comparison is indicated by blue asterisks. *P < 0.05; **P < 0.01; ***P < 0.001; ns, not significant.

We next evaluated the therapeutic efficacy and safety of circMTCO2 delivery in vivo using a tMCAO mouse model, guided by the experimental timeline shown in Fig. 7A. DiR (1,1′-dioctadecyl-3,3,3ʹ,3ʹ-tetramethylindotricarbocyanine iodide)-labeled EVs were injected intravenously at a dose of 200 μg. In vivo imaging system (IVIS) imaging demonstrated that RVG modification markedly enhanced the relative brain enrichment of EVs (Fig. 7B and Fig. S8A). TTC staining at 1 d postinjury (dpi) revealed a significant reduction in infarct volume in the RVG-EV^mt-RNA^-treated group compared with that in controls, indicating effective neuroprotection (Fig. 7C). Biochemical analyses revealed significant reductions in 4-HNE, MDA, and total iron levels in the RVG-EV^mt-RNA^ group, along with marked increases in GSH levels and the GSH/GSSG ratio, indicating effective suppression of oxidative stress and lipid peroxidation in the ischemic brain (Fig. 7D). Behavioral tests were performed to assess neurological function over 21 d. RVG-EV^mt-RNA^ treatment markedly improved modified neurological severity score (mNSS), beam walking performance, rotarod endurance, and corner test performance beginning at day 3 postinjury (Fig. 7E). These improvements persisted through day 21, indicating sustained functional recovery.

To assess the safety profile of RVG-EV^mt-RNA^ therapy, we performed comprehensive hematological and biochemical analyses at 7 dpi. Routine parameters, including red blood cell, white blood cell, platelet, alanine aminotransferase, aspartate aminotransferase, alkaline phosphatase, blood urea nitrogen, and creatinine, remained within normal ranges across all treatment groups (Fig. S8B). Additionally, hematoxylin and eosin (H&E) staining of the heart, liver, spleen, lung, kidney, and brain at 7 dpi revealed no histological abnormalities (Fig. 7F). Collectively, these results support that RVG-EV^mt-RNA^-mediated delivery of circMTCO2 confers neuroprotection in ischemic stroke by mitigating oxidative stress and lipid peroxidation, reducing infarct size, improving neurological outcomes, and exhibiting a favorable safety profile.

## Discussion

In this study, we uncovered a previously unrecognized role for the mitochondria-derived circMTCO2 as an intrinsic regulator of neuronal ferroptosis. By modulating mPTP opening and mtROS output, circMTCO2 preserves mitochondrial integrity and protects neurons against ischemic injury. These findings not only reveal a new axis in the interplay between mt-circRNAs and redox signaling but also position circMTCO2 as a promising therapeutic target. Moreover, by restoring this endogenous defense via a neuron-to-mitochondria RNA delivery system, we establish a proof of concept for organelle-specific circRNA therapy as a precision strategy to counter ferroptotic injury.

mt-circRNAs constitute a recently characterized subclass of regulatory RNAs whose functions remain largely undefined [23,24,28]. While many nuclear-derived circRNAs have been implicated in stroke through mechanisms such as microRNA sponging or transcriptional regulation, circMTCO2 is among the first mitochondria-encoded circRNAs shown to functionally regulate neuronal redox homeostasis. Mitochondria play a central role in ischemic brain injury, as cerebral ischemia leads to excessive ROS production, calcium dysregulation, and iron overload in neurons, all contributing to the development of ferroptosis [29]. Prior studies have described cytosolic circRNAs, such as circPHKA2, that indirectly regulate oxidative stress via antioxidant gene networks [22]. In contrast, our study reveals a previously unrecognized mechanism: an mt-circRNA that modulates ferroptosis from within the organelle. Notably, our results demonstrate that circMTCO2 expression is dynamically down-regulated in response to cerebral ischemia, both in vitro and in vivo. This stress-responsive behavior supports its role as an intrinsic mitochondrial safeguard, further advancing the emerging concept of mt-circRNAs as spatially precise regulators of redox signaling. Our findings, aligned with studies on non-neuronal systems like circSCAR, which inhibits mPTP opening and suppresses mtROS release in human liver cells during metabolic stress [25], suggest that organelle-localized circRNAs represent a broader built-in cellular defense mechanism against pathological ROS accumulation. By demonstrating circMTCO2’s suppression of ferroptosis via mitochondrial permeability modulation, we highlight the mechanistic and therapeutic significance of mt-circRNAs in redox biology and cell death regulation. Future studies using submitochondrial fractionation or engineered ascorbate peroxidase-based proximity labeling will be valuable to precisely map the suborganelle localization of delivered circMTCO2, providing a deeper understanding of its exact distribution and function within mitochondria.

A key mechanistic insight from this study is that circMTCO2 exerts its neuroprotective effect by interacting with ANT1, a key regulator associated with mPTP opening located in the inner mitochondrial membrane [30,31]. In ischemic neurons, mPTP opening leads to loss of mitochondrial membrane potential, excessive ROS release, and subsequent cell death. Our findings demonstrate that circMTCO2 binds ANT1 and functionally suppresses mPTP opening, thereby limiting mtROS efflux and preserving mitochondrial function. Moreover, we showed that mutation of the ANT1-binding site on circMTCO2 abolished its interaction with ANT1 and eliminated its protective effect. These findings provide direct functional evidence that the circMTCO2–ANT1 axis is essential for neuroprotection. Similar regulatory mechanisms have been described in other contexts, such as the viral protein latent membrane protein 1 that binds ANT1 to inhibit mPTP opening [32]. It is plausible that circMTCO2, by tethering to ANT1, hinders its conformational switch toward the pore-open state, thereby disrupting the feed-forward cycle of oxidative stress, mPTP opening, and ferroptotic progression. However, the precise molecular basis by which circMTCO2 inhibits mPTP opening remains to be determined. Future studies should clarify whether circMTCO2 modulates ANT1 conformation, stress-associated oligomerization, or its interaction with other regulatory partners and should also define the ANT1-side binding interface using purified or reconstituted ANT1, mutagenesis, and structural or biochemical approaches. In addition, systematic profiling of the circMTCO2 interactome in different cellular contexts will be important to determine whether additional binding partners contribute to its activity.

Importantly, this regulatory function may extend beyond ferroptosis. In ischemic stroke and other neurological disorders, mPTP opening facilitates the release of mitochondrial damage-associated molecular patterns, such as mitochondrial DNA, adenosine triphosphate, and cytochrome c, which activate inflammatory signaling and exacerbate secondary injury [33,34]. By maintaining pore closure, circMTCO2 could potentially mitigate these pro-inflammatory responses. Furthermore, since mPTP opening is involved in multiple forms of cell death, including apoptosis and necrosis [35], the circMTCO2–ANT1 axis may play a broader role in regulating cell fate decisions. While our current study focused on ferroptosis and our multiassay approach, including C11-BODIPY, DCFH-DA, MitoSOX, MitoPeDPP, 4-HNE, MDA, iron quantification, and erastin sensitivity, provides strong evidence for its involvement, we did not investigate other cell death pathways such as apoptosis or necroptosis in detail. Future studies should therefore incorporate ferroptosis inhibitors such as liproxstatin-1 or ferrostatin-1, together with pathway-specific inhibitors such as zVAD-FMK for apoptosis and necrostatin-1s for necroptosis, as well as canonical markers including cleaved caspase-3, phosphorylated mixed lineage kinase domain-like protein, and apoptosis-inducing factor translocation, to better define the cell death pathways involved.

From a translational perspective, our study introduces a dual-targeting EV system, RVG-EV^mt-RNA^, designed to enhance the delivery of circMTCO2 toward neuronal mitochondria in the ischemic brain. EVs, ranging from 30 to 180 nm, are natural intercellular messengers with the unique ability to cross biological barriers, including the blood–brain barrier [36]. Compared with viral vectors and synthetic delivery systems such as liposomes or polymeric micelles, EVs offer several important advantages, including low immunogenicity, intrinsic biocompatibility, and amenability to surface engineering [37,38]. As biotherapeutic platforms, EVs integrate the functions of carrier, therapeutic payload, and targeting moiety [39]. Building on our previous findings that RVG-labeled EVs enhance brain targeting [40,41], we further refined this strategy to achieve dual targeting. The RVG peptide promotes neuronal uptake through acetylcholine receptor binding, while the TPP–PDL moiety exploits mitochondrial membrane potential to facilitate mitochondrial delivery of circRNA cargo. TPP has long been used to functionalize mitochondria-targeting drugs, such as doxorubicin and coenzyme Q, which accumulate within mitochondria and have shown favorable safety profiles [42]. Thus, our organ-to-organelle delivery strategy is intended to address sequential delivery barriers, including brain entry, neuronal uptake, and mitochondrial localization, and our current data support functionally meaningful enhancement of this delivery process.

Nonetheless, several limitations warrant further exploration. First, while neuroprotection was observed with a single dose of RVG-EV^mt-RNA^ administered 4 h after tMCAO, the optimal therapeutic window and the potential need for repeated dosing remain unclear. It will be important to determine whether circMTCO2 retains efficacy when delivered during the subacute or chronic phases of stroke. In addition, although behavioral improvement persisted through day 21, longer-term follow-up with additional chronic outcome measures would further strengthen the translational relevance of this approach. Second, although our findings support an ANT1-centric mechanism, circRNAs often exhibit pleiotropic functions. circMTCO2 may interact with other RNA-binding proteins or microRNAs, contributing to its broader activity. Defining these additional mechanisms will be important for understanding both its therapeutic benefits and potential off-target effects. Third, the current data support enhanced brain enrichment and functionally meaningful mitochondrial delivery of RVG-EV^mt-RNA^, but more refined quantification of cell-type specificity, intramitochondrial delivery efficiency, and absolute tissue distribution in vivo will require advanced cell-resolved, suborganelle-level, and tracer-based methodologies. Fourth, future studies should evaluate efficacy and safety in female cohorts to determine whether sex-dependent differences influence therapeutic responses. Finally, in vivo loss-of-function investigation of endogenous circMTCO2 remains technically challenging. The development of mitochondria-accessible, junction-targeting knockdown strategies will therefore be an important future direction, not only for the mechanistic dissection of circMTCO2 but also for the therapeutic suppression of pathogenic mt-circRNAs more broadly.

## Conclusion

Our study identifies circMTCO2 as a mitochondria-derived circRNA that protects neurons from ischemic injury by modulating mPTP-dependent redox balance. The development of a dual-targeting EV platform represents an important advance in spatially resolved RNA therapeutics. Together, our findings illuminate a novel layer of mitochondrial redox regulation and lay the groundwork for a new class of noncoding RNA-based interventions for ischemic stroke and other redox-driven neurodegenerative disorders. More broadly, these findings support the concept that organ-to-organelle circRNA delivery can be leveraged as a precision therapeutic strategy for ischemic stroke.

## Materials and Methods

### Animals and model of tMCAO

Male C57BL/6 mice, aged 8 to 9 weeks and weighing 20 to 22 g, were used in this study. All animal procedures were approved by the Animal Care and Use Committee (No. 202201013). Mice were anesthetized with an intraperitoneal injection of sodium pentobarbital (50 mg/kg). The right common carotid artery, external carotid artery, and internal carotid artery were exposed. A silicon-coated monofilament (diameter 0.20 to 0.21 mm) was inserted through the external carotid artery and advanced into the internal carotid artery to occlude the middle cerebral artery. CBF was continuously monitored using laser Doppler flowmetry (RWD Life Science, China), with successful occlusion confirmed by a >70% reduction in baseline CBF. After 60 min of occlusion, the filament was withdrawn to allow reperfusion. Sham surgery was performed with identical procedures, except for the insertion of the monofilament. All procedures followed the ARRIVE guidelines for animal research. Mice were randomly assigned to groups, and sample sizes were determined based on prior experience to ensure statistical power. Data analysis was conducted in a double-blinded manner with no exclusions.

### MDA assay

MDA levels in mouse brain were quantified using an MDA assay kit (Solarbio Life Science, China). Brain tissues were homogenized in lysis buffer and centrifuged, and 100 μl of the supernatant was mixed with 300 μl of MDA detection solution. The mixture was incubated at 100 °C for 60 min, and optical density (OD) was measured at 532 nm using a microplate reader. The MDA content was calculated using the following formula according to the manufacturer’s instructions: MDA content (nmol/mg protein) = (53.763 × Δ*A*)/*C*_pr_, where Δ*A* = Δ*A*_532_ − Δ*A*_600_, Δ*A*_532_ = *A*_532T_ − *A*_532B_, and Δ*A*_600_ = *A*_600T_ − *A*_600B_. *A*_532T_ and *A*_600T_ are the sample absorbances at 532 and 600 nm, respectively, and *A*_532B_ and *A*_600B_ are the blank absorbances. *C*_pr_ is the protein concentration.

### 4-HNE assay

4-HNE levels in mouse brain were analyzed using a 4-HNE assay kit (Abcam, UK). Brain tissues were lysed with radioimmunoprecipitation assay (RIPA) lysis buffer and centrifuged to collect the supernatant. A 50-μl standard or sample was added to a 4-HNE conjugate-coated plate and incubated for 10 min. After adding 50 μl of diluted anti-4-HNE antibody, the plate was incubated for 1 h. After 3 washes with 1× wash buffer, 100 μl of the diluted secondary antibody–horseradish peroxidase (HRP) conjugate was added, followed by another 1-h incubation. After another wash, 100 μl of substrate solution was added and incubated for 15 min. The reaction was stopped with 100 μl of stop solution, and OD was measured at 450 nm. 4-HNE concentration was determined using a standard curve derived from the logarithmic concentration and OD_450_ values.

### GSH and GSSG assays

GSH and GSSG levels in mouse brain were measured using GSH and GSSG assay kits (Solarbio Life Science, China) following the manufacturer’s instructions. Brain tissues were homogenized in lysis buffer, and the supernatant was collected. Standard solutions of GSH and GSSG were prepared at various concentrations. GSSG content was calculated as follows: GSSG content (μmol/g tissue) = GSSG concentration × *V*/*W* × 612.62, where *V* is the total supernatant volume and *W* is the tissue wet weight. Similarly, GSH content was calculated using GSH content (μmol/g tissue) = GSH concentration × *V*/*W* × 307.33.

### Iron assay

Iron levels were measured using an iron assay kit (Solarbio Life Science, China). Brain tissues were homogenized in extraction solution, and the lysates were centrifuged to collect the supernatant. A total of 120 μl of the supernatant was mixed with 60 μl of Reagent 1 and 120 μl of Reagent 2 and then incubated at 100 °C for 5 min. After incubation, 60 μl of chloroform was added, and the mixture was centrifuged. The upper inorganic phase was transferred to a 96-well plate, and OD was measured at 532 nm. The total iron content was calculated according to the assay kit instructions.

### Cell culture and OGD with reoxygenation

Mouse neuroblastoma N2a cells were obtained from the National Infrastructure of Cell Line Resource and cultured in minimum essential medium supplemented with 10% fetal bovine serum and 1% penicillin/streptomycin (Thermo Fisher Scientific, USA). Cells were maintained at 37 °C in a 5% CO_2_ humidified incubator and passaged every 3 d. For OGD, N2a cells were cultured in glucose-free medium and exposed to hypoxia for 3 h in a chamber with a gas mixture of 95% N_2_ and 5% CO_2_. After OGD, cells were returned to normoxic conditions (5% CO_2_) and complete glucose-containing medium for 24 h of reoxygenation to model ischemia/reperfusion injury in vitro. OGD/reoxygenation was used as an in vitro ischemia/reperfusion paradigm to examine intrinsic mitochondrial dysfunction and ferroptosis-related responses under controlled conditions. Unless otherwise specified, OGD refers to a 3-h OGD followed by 24-h reoxygenation.

### Drug treatments

To induce ferroptosis, cells were treated with 40 μM erastin (MedChemExpress, USA) for 24 h. To modulate mitochondrial function, cells were pretreated for 2 h with either 1 μM Mito-T, a mitochondria-targeted antioxidant, or 10 μM CSA (MedChemExpress, USA), an inhibitor of the mPTP. To assess treatment efficacy, N2a cells were incubated in a 24-well plate with 40 μg of either EV^Ctrl^, RVG-EV^RNA^, or RVG-EV^mt-RNA^ for 24 h.

### Cell viability assay and Live/Dead cell staining

Cell viability was assessed using CCK-8 (Beyotime, China). N2a cells were seeded into a 96-well plate and cultured for 24 h at 37 °C in a humidified incubator with 5% CO_2_. After the indicated treatments, cells were washed with phosphate-buffered saline (PBS) and incubated with fresh culture medium containing 10% (v/v) CCK-8 reagent. After 3 h of incubation at 37 °C, the absorbance at 450 nm was measured using a microplate reader (Multiskan FC, Thermo Fisher Scientific, USA). For Live/Dead cell staining, N2a cells were seeded into 24-well plates and treated as indicated. Live and dead cells were stained using the Live/Dead Viability/Cytotoxicity Kit (Beyotime, China) according to the manufacturer’s instructions. Briefly, cells were incubated with calcein-AM and propidium iodide, and fluorescence images were acquired by fluorescence microscopy. The percentage of live cells was quantified from 5 random fields per well.

### Measurement of ROS and lipid peroxidation

C11-BODIPY 581/591 (Thermo Fisher Scientific, USA) was used to measure intracellular lipid peroxidation; DCFH-DA (MedChemExpress, USA), intracellular ROS; MitoPeDPP (Dojindo Laboratories, Japan), mitochondrial lipid peroxidation; and MitoSOX (Thermo Fisher Scientific, USA), mtROS. C11 BODIPY 581/591 was diluted to 10 μM, DCFH-DA to 5 μM, MitoSOX to 5 μM in serum-free medium, and MitoPeDPP to 0.5 μM in Hank’s solution with 10 mM HEPES. After treatment, N2a cells were incubated with the corresponding probes at 37 °C for 30 min in the dark, followed by nuclear staining with 4′,6-diamidino-2-phenylindole (DAPI). Images were captured using a fluorescence microscope (BX53, Olympus, Japan). Fluorescence excitation/emission wavelengths were as follows: C11-BODIPY 581/591, 581/591 nm (reduced) and 488/510 nm (oxidized); DCFH-DA, 488/525 nm; MitoPeDPP, 470/525 nm; MitoSOX, 396/610 nm; and DAPI, 405/461 nm. The fluorescence intensity was quantified using the ImageJ software by measuring the mean gray value (average fluorescence intensity) within defined regions of interest, across at least 5 randomly selected fields per group. Unless otherwise specified, this method was applied for cell fluorescence quantification throughout the study.

### mPTP assay

mPTP opening was assessed using a calcein–CoCl_2_ quenching assay kit (Beyotime, China) following the manufacturer’s instructions. N2a cells were incubated with a 1× calcein-AM and 1× CoCl_2_ fluorescence quenching solution for 40 min at 37 °C. Calcein was excited at 494 nm, and emission was recorded at 517 nm. Fluorescence images were captured using a fluorescence microscope, and intensity was quantified using the ImageJ software.

### Mitochondrial transmembrane potential assay

Mitochondrial transmembrane potential was assessed using the lipophilic cation probe JC-1 (MedChemExpress, USA), which accumulates in a potential-dependent manner. A decrease in the red (JC-1 aggregates) to green (JC-1 monomers) fluorescence intensity ratio indicates mitochondrial depolarization. Cells were incubated with 2 μM JC-1 solution for 30 min at 37 °C in the dark. After resuspension in ice-cold PBS, cells were analyzed by flow cytometry (FACSAria III, Becton Dickinson, USA), collecting data from at least 100,000 cells. The fluorescence excitation/emission wavelengths were as follows: JC-1 monomer, 510/527 nm (green), and JC-1 aggregate, 585/590 nm (red). The red/green fluorescence ratio was used to quantify mitochondrial dysregulation.

### CircRNA sequencing

Total RNA was extracted from mouse brain tissue using RNAiso Plus (Takara Bio Inc., Japan). Ribosomal RNA (rRNA) was removed using the KAPA RNA HyperPrep Kit with RiboErase for Illumina (HMR) (Kapa Biosystems, Inc., USA). PolyA tailing was performed on the rRNA-depleted RNA, followed by purification with CleanNGS magnetic beads (GC Biotech, the Netherlands). An RNase R reaction was then conducted at 37 °C for 30 min, and the RNA was purified again. Rolling circle reverse transcription was performed using the SMARTer PCR cDNA Synthesis Kit (Takara Bio Inc., Japan), and cDNA amplification was carried out with the KAPA HotStart mix (Kapa Biosystems, USA). Fragments between 200 and 2,000 bp were selected for sequencing. End repair and dA-tailing were performed using the NEBNext Ultra II End Repair/dA-Tailing Module (NEB, USA). Libraries were loaded onto MinION Mk1B flow cells and sequenced on the MinION platform (Oxford Nanopore Technologies, UK) using standard sequencing buffers. Full-length circRNAs were detected using CIRI-long as previously reported [43].

### gDNA extraction

gDNA was extracted from the mouse cerebral cortex using the MiniBEST Universal Genomic DNA Extraction Kit (Takara Bio Inc., Japan) according to the manufacturer’s protocol. The DNA concentration was measured with a NanoDrop One Microvolume spectrophotometer (Thermo Fisher Scientific, USA).

### RNA isolation and qRT-PCR

Total RNA from cells and tissues was extracted using the TRIzol method (Thermo Fisher Scientific, USA). cDNA synthesis was performed with PrimeScript RT Master Mix (Takara Bio Inc., Japan). qRT-PCR was conducted using TB Green Premix Ex Taq II (Takara Bio Inc., Japan), with primers designed via National Center for Biotechnology Information Primer-BLAST (detailed in Table S1).

### RNase R and Act D treatment

For RNase R treatment, 2 μg of RNA was incubated at 37 °C for 5 min with or without 3 U/μg RNase R (LGC Biosearch Technologies, UK). Reverse transcription was performed using random hexamer primers, followed by qRT-PCR. For Act D treatment, N2a cells were treated with 5 μg/ml Act D (MedChemExpress, USA) to inhibit transcription and collected at specified time points. RNA was extracted, and the stability of circRNA and linear mRNA was analyzed by qRT-PCR.

### Plasmid construction

Plasmids for overexpressing circ MTCO2 were constructed using the pLO5-ciR vector, while GFP-tagged circMTCO2 plasmids were generated with the pLC5-ciR vector. A mutant plasmid of circ MTCO2, featuring a deletion between positions 36 and 120 nt, was also constructed (GenScript, China). The ANT1 plasmid, designed to express the ANT1 protein, was created by cloning the coding sequence of the mouse *SLC25A4* gene (NM_007450.5) into the pCDNA3.1(+) vector (GenScript, China). All plasmids were confirmed by DNA sequencing and transfected using Lipofectamine 3000 Transfection Reagent (Thermo Fisher Scientific, USA).

### EV engineering and characterization

Suspension 293F cells (Thermo Fisher Scientific, USA) were cultured in Nalgene single-use Erlenmeyer flasks on an orbital shaker at 100 rpm. 293F cells were cotransduced with lentiviruses expressing RVG–Lamp2b and circMTCO2, with or without GFP (OBiO Tech, Inc., China). Forty-eight hours posttransduction, hygromycin B and puromycin (MedChemExpress, USA) were added to the culture medium to establish a stable cell line, which was passaged every 3 d. EV^Ctrl^ and RVG-EV^RNA^ were harvested from the supernatant of untransduced 293F cells and the stable cell line via sequential ultracentrifugation. The culture medium was centrifuged at 500 × g for 10 min to remove cells and at 3,000 × g for 10 min to remove cell debris and underwent ultracentrifugation at 100,000 × g for 2 h at 4 °C using Avanti JXN-30 (Beckman Coulter, USA). To ensure sterilization, the resuspended EVs were filtered with a 0.22-μm filter. To produce RVG-EV^mt-RNA^, the mitochondria-targeting moiety TPP–PDL (Ruixibio, China) was loaded into RVG-EV^RNA^. Electroporation was performed with 0.5 μM TPP–PDL in 4-mm electroporation cuvettes at 700 V (Bio-Rad Laboratories, USA), and the samples were placed on ice for 30 min. EVs were then centrifuged at 100,000 × g for 1 h at 4 °C to collect RVG-EV^mt-RNA^. For RVG-EV^mt-mut RNA^, the lentivirus expressing circMTCO2 was replaced with one expressing the mutant circMTCO2 (36 to 120 nt), and the procedure followed was identical to that for RVG-EV^mt-RNA^. The EV protein concentration was measured using the Pierce BCA Protein Assay Kit (Thermo Fisher Scientific, USA). EV size distributions (intensity-weighted mode) were determined by dynamic light scattering using Zetasizer Ultra (Malvern Panalytical, UK). Transmission electron microscopy was performed as previously described [44].

### Western blotting

Samples were lysed in RIPA buffer, separated by 10% sodium dodecyl sulfate–polyacrylamide gel electrophoresis and transferred to a polyvinylidene fluoride membrane. The blots were incubated with primary antibodies overnight at 4 °C, followed by HRP-conjugated secondary antibodies. Detection was performed using Super ECL Plus (Applygen Technologies Inc., China), and the signal intensity was quantified with the ChemiDoc MP Imaging System (Bio-Rad Laboratories, USA). The antibodies used are listed in Table S2, with glyceraldehyde-3-phosphate dehydrogenase serving as the loading control.

### Absolute qPCR

Absolute qPCR was performed to determine the copy number of circMTCO2 in EVs. A 352-bp fragment containing the backspliced junction of circMTCO2 was inserted into the pcDNA3.1(+) vector (GenScript, China). Serial 10-fold dilutions of the plasmid were used to generate a standard curve through real-time PCR. The copy number was calculated using the following formula: copy number (copy/μl) = 6.02 × 10^23^ × plasmid concentration (ng/μl) × 10^−9^/[(molecular weight of vector + molecular weight of inserted fragment) × 660]. The standard curve equation was derived as *Y* = *aX* + *b*, where *X* is the log-transformed initial copy number and *Y* is the corresponding cycle threshold value. The cycle threshold values of circMTCO2 were used to determine its copy number in EVs by applying the standard curve equation.

### Evaluation of the mitochondrial targeting property

To evaluate mitochondrial targeting, N2a cells were incubated with 40 μg of RVG-EV^RNA-GFP^ or RVG-EV^mt-RNA-GFP^ in a 24-well plate for 24 h. The cells were then stained with 500 nM MitoTracker Red (Thermo Fisher Scientific, USA) in serum-free culture medium for 40 min at 37 °C in the dark. After 3 washes with PBS, nuclei were stained with DAPI. The fluorescence excitation/emission wavelengths were as follows: MitoTracker Red, 581/644 nm; GFP, 488/509 nm; and DAPI, 405/461 nm. Images were acquired using a Zeiss LSM770 confocal microscope (Zeiss ZEN 2 software).

### Mitochondria isolation

N2a cells cultured in 10-cm dishes were washed twice with ice-cold PBS and detached using a cell scraper. The cell pellet was resuspended in ice-cold IB-1 homogenization buffer (225 mM mannitol, 75 mM sucrose, 0.1 mM EGTA, and 30 mM Tris–HCl) containing a 1× protease inhibitor cocktail (Beyotime, China). Homogenization was performed with 90 strokes in a Dounce homogenizer (Sigma-Aldrich, USA). The homogenate was centrifuged at 740 × g for 5 min at 4 °C to collect the supernatant, which was then centrifuged at 9,000 × g for 10 min at 4 °C to isolate crude mitochondria. The mitochondrial pellet was resuspended in ice-cold IB-2 buffer (225 mM mannitol, 75 mM sucrose, and 30 mM Tris–HCl) and purified by centrifugation at 10,000 × g for 10 min at 4 °C. The purified mitochondria were stored at −80 °C.

### RNA pull-down assay

RNA pull-down assays were performed using the Magnetic RNA-Protein Pull-Down Kit (Thermo Fisher Scientific, USA) according to the manufacturer’s instructions. N2a cells were cross-linked with 1% formaldehyde for 10 min. The cells were then lysed in Pierce IP Lysis Buffer (Thermo Fisher Scientific, USA), supplemented with 2 mmol/l dithiothreitol (Solarbio Life Science, China), 1 mM phenylmethylsulfonyl fluoride (Beyotime, China), and 200 units/μl RiboLock RNase Inhibitor (Thermo Fisher Scientific, USA) for 20 min on ice. Lysates were sonicated for 3 min using JY92-IIN Ultrasonic Homogenizer. The lysates were incubated overnight at 37 °C with 100 pmol of 3′-biotin-labeled circMTCO2 probe or a negative control probe (Genepharma, China) (detailed in Table S1). Following incubation, 50 μl of Dynabeads M-280 streptavidin (Thermo Fisher Scientific, USA) was added, and the mixture was rotated end to end at room temperature for 1 h. The beads were washed 5 times with wash buffer. Proteins pulled down by the probes were analyzed by Western blotting or LC–MS/MS.

LC–MS/MS analysis was performed using ORBITRAP ECLIPSE Mass Spectrometer (Thermo Fisher Scientific, USA). Peptides were loaded onto a C18 trap column, connected to a C18 reverse-phase analytical column, and eluted over a 60-min gradient (6% to 95% acetonitrile, 0.1% formic acid in water) at a flow rate of 300 nl/min. MS1 spectra were acquired at 120,000 resolution (350 to 1,500 *m*/*z*), and precursor ions were selected for MS2 analysis with higher-energy collisional dissociation collision energy (33%). Spectra were acquired at 15,000 resolution (first mass 110 *m*/*z*).

To identify the binding region between circMTCO2 and ANT1, truncated circMTCO2 probes of varying lengths were synthesized by GenScript (see Table S1 for details). In an in vitro RNA–protein pull-down assay, 50 pmol of the truncated circMTCO2 probes were incubated with mitochondria isolated from ANT1-overexpressed N2a cells, followed by coincubation with Dynabeads M-280 streptavidin, as previously described. Western blotting was performed to detect the bound proteins.

### RNA immunoprecipitation

RIP was performed using the Magna RIP RNA-Binding Protein Immunoprecipitation Kit (Merck, Germany). N2a cells were washed with ice-cold PBS and lysed in Pierce IP Lysis Buffer containing a protease inhibitor and an RNase inhibitor (Thermo Fisher Scientific, USA) for 20 min on ice. Protein A/G magnetic beads (Thermo Fisher Scientific, USA) were incubated with antibodies against ANT1 (ABclonal, China) or control rabbit immunoglobulin G (Cell Signaling Technology, USA) for 1 h at room temperature. The cell lysate supernatant was then incubated with the antibody–bead complexes overnight at 4 °C. After immunoprecipitation, the complexes were washed 5 times with wash buffer. Protein digestion was carried out with proteinase K buffer at 55 °C for 30 min. RNA was extracted using the TRIzol method and analyzed by qRT-PCR.

### FISH and immunofluorescence staining

RNA FISH was performed to detect circMTCO2 using the Fluorescent In Situ Hybridization Kit (RiboBio, China) according to the manufacturer’s instructions. Mitochondria in N2a cells were stained with 400 nM MitoTracker Deep Red (Thermo Fisher Scientific, USA) for 1 h, followed by washing with PBS. Cells were fixed with 4% formaldehyde for 15 min at room temperature and permeabilized with 0.05% Triton X-100 for 10 min at 4 °C. After 30 min of prehybridization at 37 °C, hybridization was performed overnight at 60 °C in a dark, humid chamber with 20 nM digoxin-labeled oligonucleotide probe targeting the circMTCO2 backsplice sequence (SinoGenoMax Technologies, China). Cells were washed with 4×, 2×, and 1× saline-sodium citrate (Beyotime, China) and incubated with anti-digoxigenin–fluorescein isothiocyanate (FITC) antibody (1:300) (Roche, Switzerland) for 1 h at room temperature.

To assess colocalization of circMTCO2 and ANT1 protein, cells were incubated with an ANT1 antibody (ABclonal, China) for 4 h at room temperature. After 3 PBS washes, cells were incubated with Alexa Fluor 594 goat anti-rabbit immunoglobulin G secondary antibody (Thermo Fisher Scientific, USA) for 1 h. Nuclei were stained with DAPI, and slides were mounted with ProLong Gold antifade reagent (Thermo Fisher Scientific, USA). The fluorescence excitation/emission wavelengths were as follows: anti-digoxigenin–FITC, 488/517 nm, and Alexa Fluor 594, 594/615 nm. Images were captured using a Leica MICA microscope (Software version: Leica LAS X 3.10.0).

### Prediction of the RNA secondary structure and RNA–protein interactions

The secondary structure of circMTCO2 was predicted using the RNAfold web server, part of the ViennaRNA Web Services (Institute for Theoretical Chemistry, University of Vienna), based on the ViennaRNA Package [45]. Predictions were generated using the minimum free energy model, with color scales indicating the confidence level of each base-pair prediction; red shades denoted regions of high prediction confidence (website: http://rna.tbi.univie.ac.at/). To predict potential interaction sites between circMTCO2 and ANT1, we utilized the catRAPID omics platform (version 2.1).

### IVIS spectrum for fluorescence imaging

EVs were labeled with DiR as previously described [44]. DiR-labeled EVs (200 μg) were intravenously administered into mice via the tail vein. Fluorescence imaging of organs was performed at 4 h postinjection using a small-animal imaging system (Xenogen IVIS Spectrum, PerkinElmer, USA) with excitation and emission wavelengths of 745 and 800 nm, respectively. IVIS was used as a semiquantitative method to compare relative biodistribution among EV formulations under standardized conditions. Regions of interest were defined, and the average radiant efficiency was quantified in (p/s/cm^2^/sr)/(μW/cm^2^).

### TTC staining

Mice were sacrificed 24 h post-tMCAO injury, and their brains were quickly harvested and sectioned into 1-mm-thick coronal slices. The tissue slices were incubated in 2% TTC (Merck, Germany) at 37 °C for 20 min, followed by fixation in 4% paraformaldehyde. Infarct areas appeared white, while viable tissue stained red. The infarction percentage was calculated using the following formula: Infarct area (%) = [(contralateral hemisphere area − non-infarct area of the ipsilateral hemisphere)/contralateral hemisphere area] × 100. Because all coronal sections were 1 mm thick, infarct area summed across sections was expressed as infarct volume. Images were analyzed using the ImageJ software (National Institutes of Health, USA).

### Immunofluorescence staining

Frozen 12-μm coronal brain sections were prepared using a Leica CM1950 cryostat (Leica Biosystems, Germany). Sections were blocked with 3% bovine serum albumin and 0.3% Triton X-100 in PBS for 1 h at room temperature. After overnight incubation with primary antibodies, sections were incubated with secondary antibodies for 2 h at room temperature. Details of the antibodies used are provided in Table S2. Sections were mounted using Antifade Mounting Medium with DAPI (SouthernBiotech, USA). Images were acquired using a BX53 microscope (Olympus, Japan).

### H&E staining

Tissue samples (brain, liver, heart, lung, spleen, and kidney) were fixed in 4% paraformaldehyde, embedded in paraffin, and sectioned at 4-μm thickness using a microtome (Leica Biosystems, Germany). H&E staining was performed using an H&E staining kit (Beyotime, China). Images were captured using a BX53 microscope (Olympus, Japan).

### Hematological and biochemical analyses

Blood samples were collected from the tail vein at 7 d post-tMCAO injury. Hematological parameters, including red blood cell count, white blood cell count, and platelet count, were measured using a TEK-II Mini hematology analyzer (Tecom Science Corporation, China). Biochemical markers, including alanine aminotransferase, aspartate aminotransferase, alkaline phosphatase, blood urea nitrogen, and creatinine, were measured using a TBA-40FR biochemical analyzer (Toshiba Medical Systems Corporation, Japan).

### Neurological function assessment and behavioral tests

Mice were randomly assigned to 5 groups and pre-trained for 3 d. Testing was conducted on the baseline (presurgery) day and on postsurgery days 1, 3, 5, 7, 10, 14, and 21. Behavioral tests were performed and analyzed by 2 independent, blinded technicians. Neurological function was evaluated up to 21 d post-tMCAO using mNSS, which evaluates motor, sensory, and balance functions on a scale from 0 (normal) to 14 (most severe impairment).

For the beam walking test, mice were placed at the start of a 1-m-long, 12-mm-wide wooden beam elevated 50 cm above the ground. Performance was scored on a 6-point scale: 0 = fall off the beam; 1 = unable to cross or place the affected limb on the beam but can maintain balance; 2 = unable to cross but can place the affected limb on the beam and balance for ≥5 s; 3 = crosses <50% of the beam with missteps; 4 = crosses >50% of the beam with missteps; 5 = crosses the beam with ≤ 2 limb slips; and 6 = normal crossing without limb slips.

For the rotarod test, mice were placed on a rotarod system (Nanjing Calvin Biotechnology Co., Ltd., China) with a rod accelerating from 5 to 40 rpm over 300 s, followed by a constant speed for 2 min. The trial ended when the mouse fell off, and the latency to fall was recorded. Each trial was repeated 3 times, with a 30-min interval.

For the corner turn test, 2 wooden boards (dimensions: 30 × 20 × 1 cm) formed a 30° corner apparatus. Mice were placed 12 cm from the corner and allowed to walk into it, where the direction of turning was recorded to assess asymmetry. Each mouse participated in 10 trials. The asymmetry percentage was calculated as follows: Asymmetry (%) = (number of left turns/(number of left turns + number of right turns)) × 100%.

### Statistical analysis

Data are presented as mean ± standard error of the mean. For relative comparisons, data were normalized to the control group, as specified in the figure legends. Statistical analyses were conducted using GraphPad Prism v8.0 (GraphPad Software Inc., USA). The Shapiro–Wilk test was used to assess data normality. For normally distributed data, differences between 2 groups were analyzed using an unpaired 2-tailed Student *t* test. Comparisons among 3 or more groups were performed using one-way analysis of variance (ANOVA) or 2-way ANOVA followed by Tukey’s multiple-comparisons test, as appropriate. Statistical significance was defined as *P < 0.05, **P < 0.01, and ***P < 0.001.

## Data Availability

The data that support the findings of this study are available in the Supplementary Materials.

## Supplementary Materials

Supplementary 1 Graphical Abstract Figs. S1 to S8 Tables S1 and S2
