# Supplementary material for: Extracellular Vesicle-Mediated Delivery of Mitochondrial Circular RNA MTCO2 Protects against Cerebral Ischemia by Modulating mPTP-Dependent Ferroptosis
Source: Research (Wash D C). 2026 Apr 14;9:1232. doi: 10.34133/research.1232 (PMC13077131; doi:10.34133/research.1232)
Supplement: Supplementary 1 — Graphical Abstract Figs. S1 to S8 Tables S1 and S2 [file research.1232.f1.pdf]

Supporting Information

Extracellular vesicle-mediated delivery of mitochondrial circRNA MTCO2 protects against cerebral ischemia by modulating mPTP-dependent ferroptosis

Graphical abstract

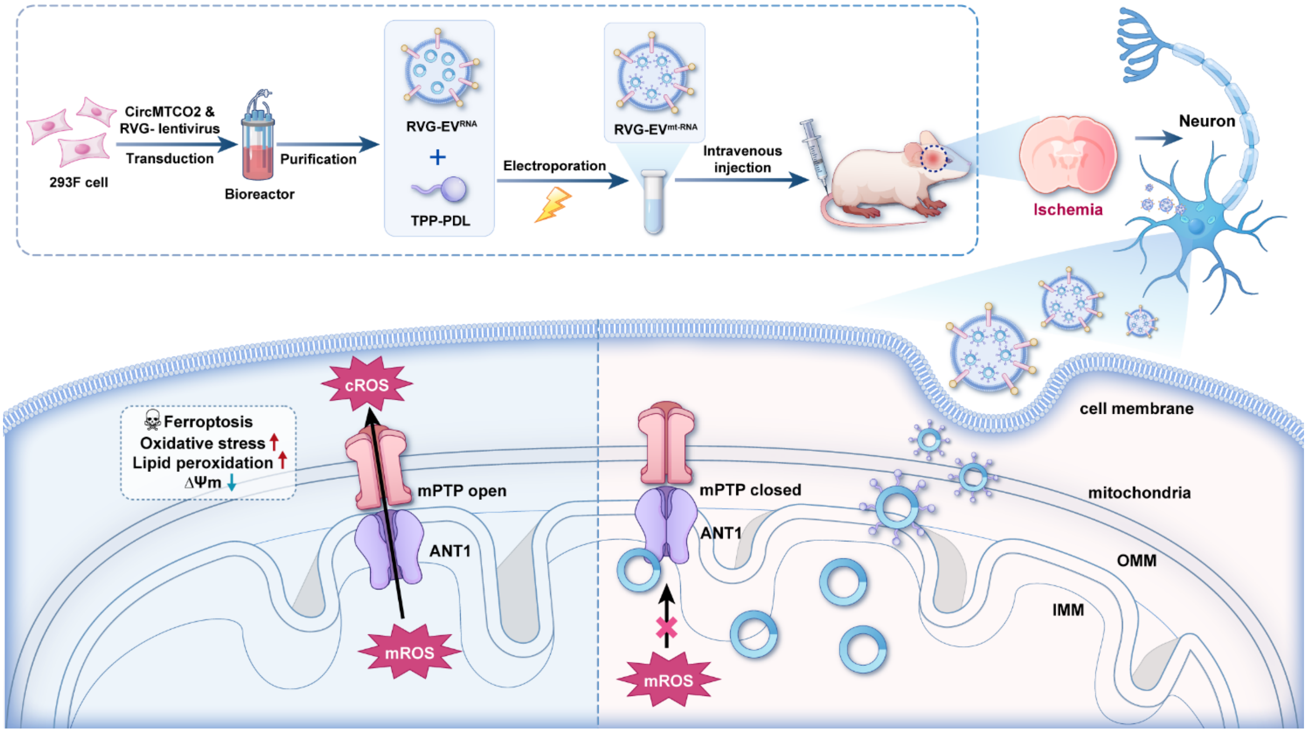

Supplementary Figures

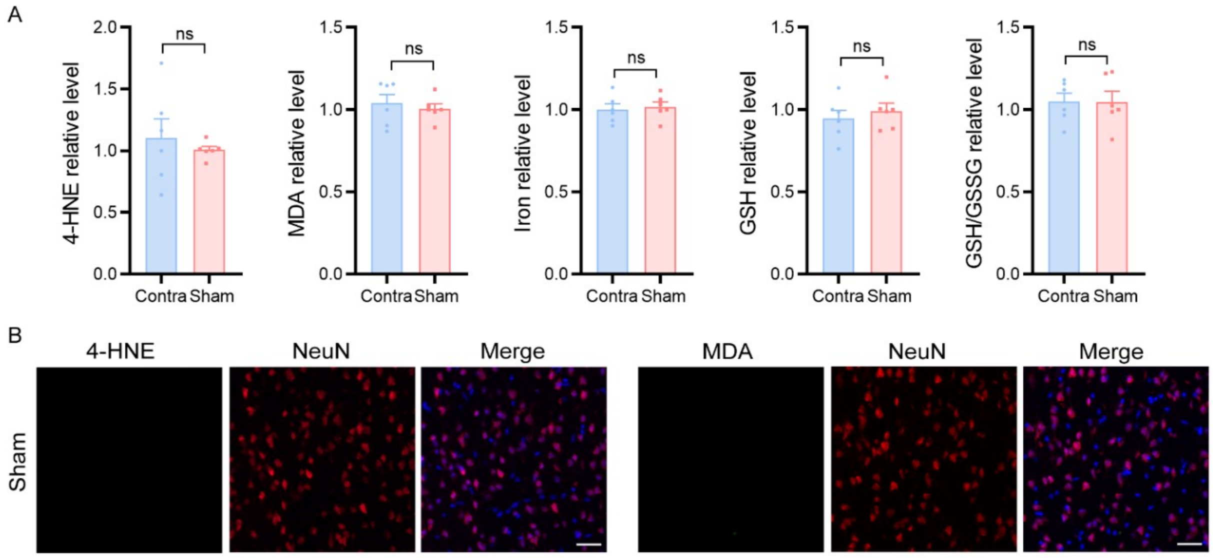

Figure S1. Control data from sham animals and contralateral hemispheres of tMCAO mice. (**A**) Quantification of 4-HNE, MDA, total iron, GSH, and the GSH/GSSG ratio in the contralateral (Contra) hemispheres of tMCAO mice at 24 hpi and the corresponding hemisphere of sham animals. (**B**) Immunofluorescence staining of 4-HNE/NeuN and MDA/NeuN in the cortex of sham mice. Scale bars, 30 μm. Data are presented as mean ± SEM (n = 6 per group). Statistical analysis was performed using an unpaired two-tailed Student's t-test. ns, not significant.

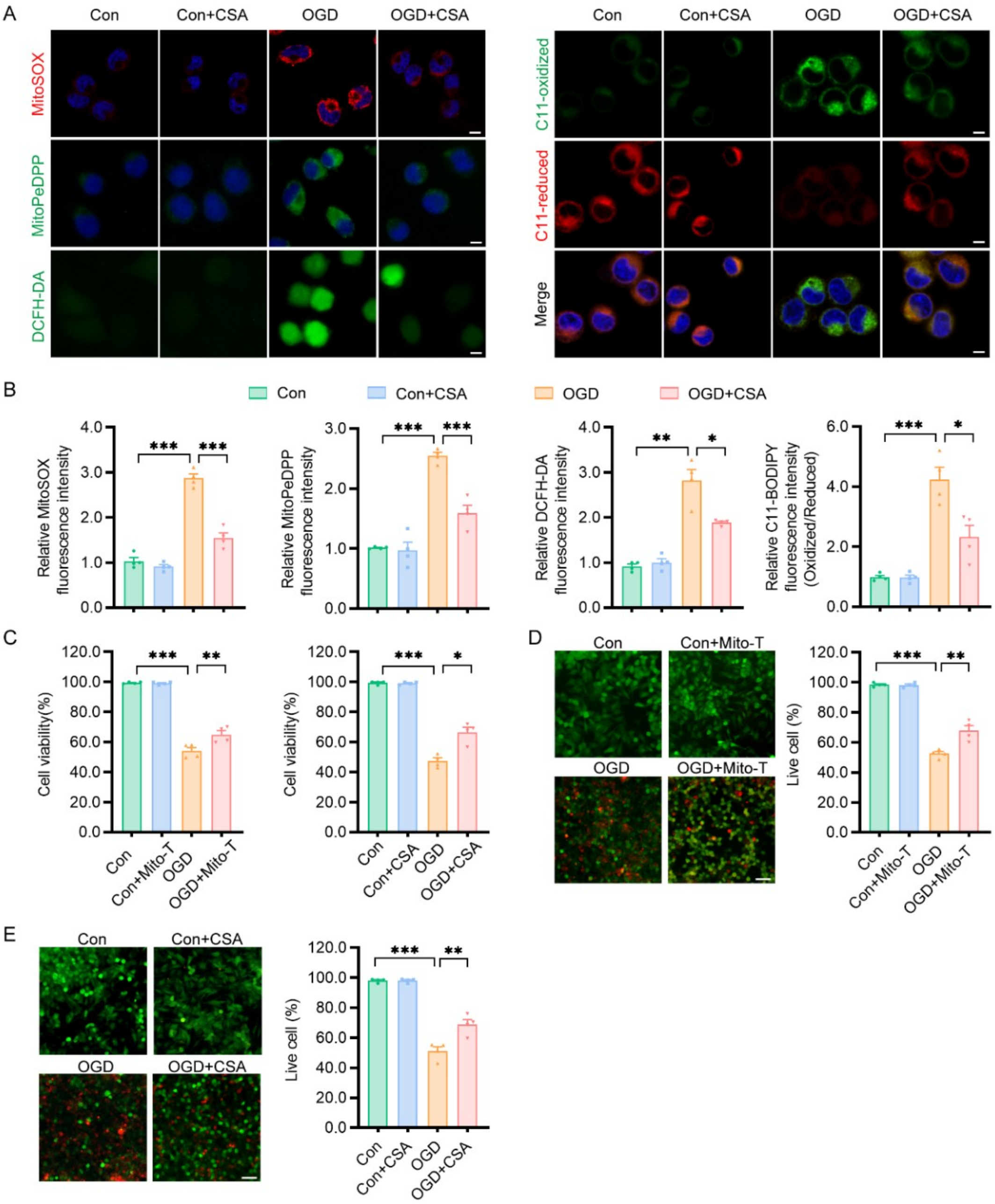

Figure S2. Inhibition of mPTP opening reduces mtROS and lipid peroxidation in OGD-treated neurons.

(**A**) Representative fluorescence images of MitoSOX (mtROS), MitoPeDPP (mitochondrial lipid peroxidation), DCFH-DA (cytosolic ROS), and C11-BODIPY (oxidized and reduced forms) in control and OGD-treated N2a cells, with or without Cyclosporin A (CSA). (**B**) Quantification of fluorescence intensity for the indicated oxidative stress markers. (**C-E**) Cell viability assessed by the Cell Counting Kit-8 (CCK-8) and Live (green) /Dead (red) cell staining in control and OGD-treated N2a cells, with or without Mito-TEMPO (Mito-T), and with or without Cyclosporin A (CSA). Scale bars, 5 μm (A), 50 μm (D,E). Data are presented as mean ± SEM (n = 4 per group). Statistical analysis was performed using an unpaired two-tailed Student's t-test. *$P < 0.05$; **$P < 0.01$; ***$P < 0.001$.

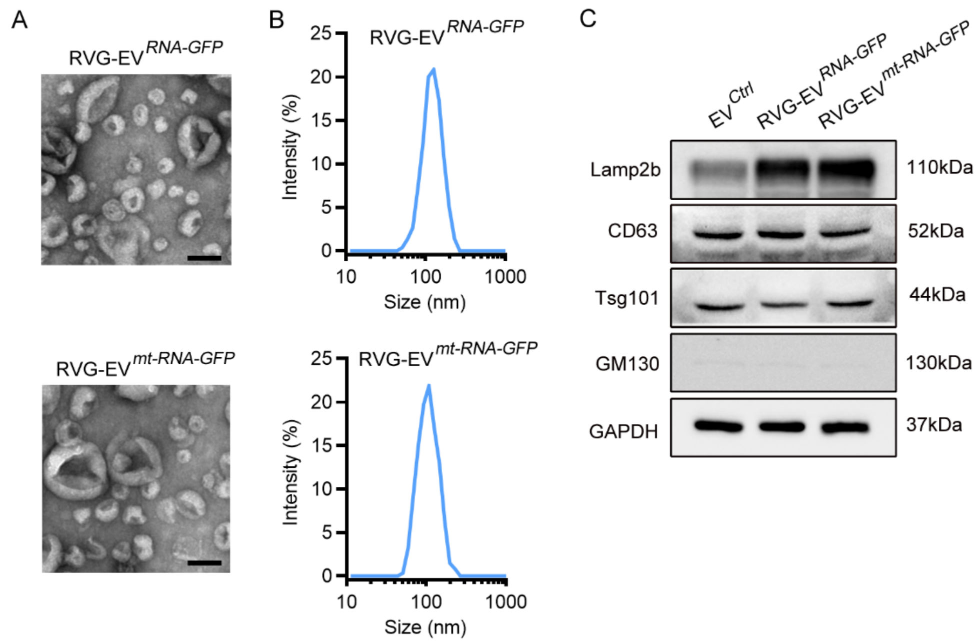

Figure S3. Characterization of RVG-EV$^{RNA-GFP}$ and RVG-EV$^{mt-RNA-GFP}$.

(**A**) Transmission electron microscopy images of RVG-EV$^{RNA-GFP}$ and RVG-EV$^{mt-RNA-GFP}$. Scale bars, 100 nm. (**B**) Nanoparticle size distribution of the indicated EVs. (**C**) Western blot of EV markers (Lamp2b, CD63, and Tsg101) and the Golgi marker (GM130) in purified EVs.

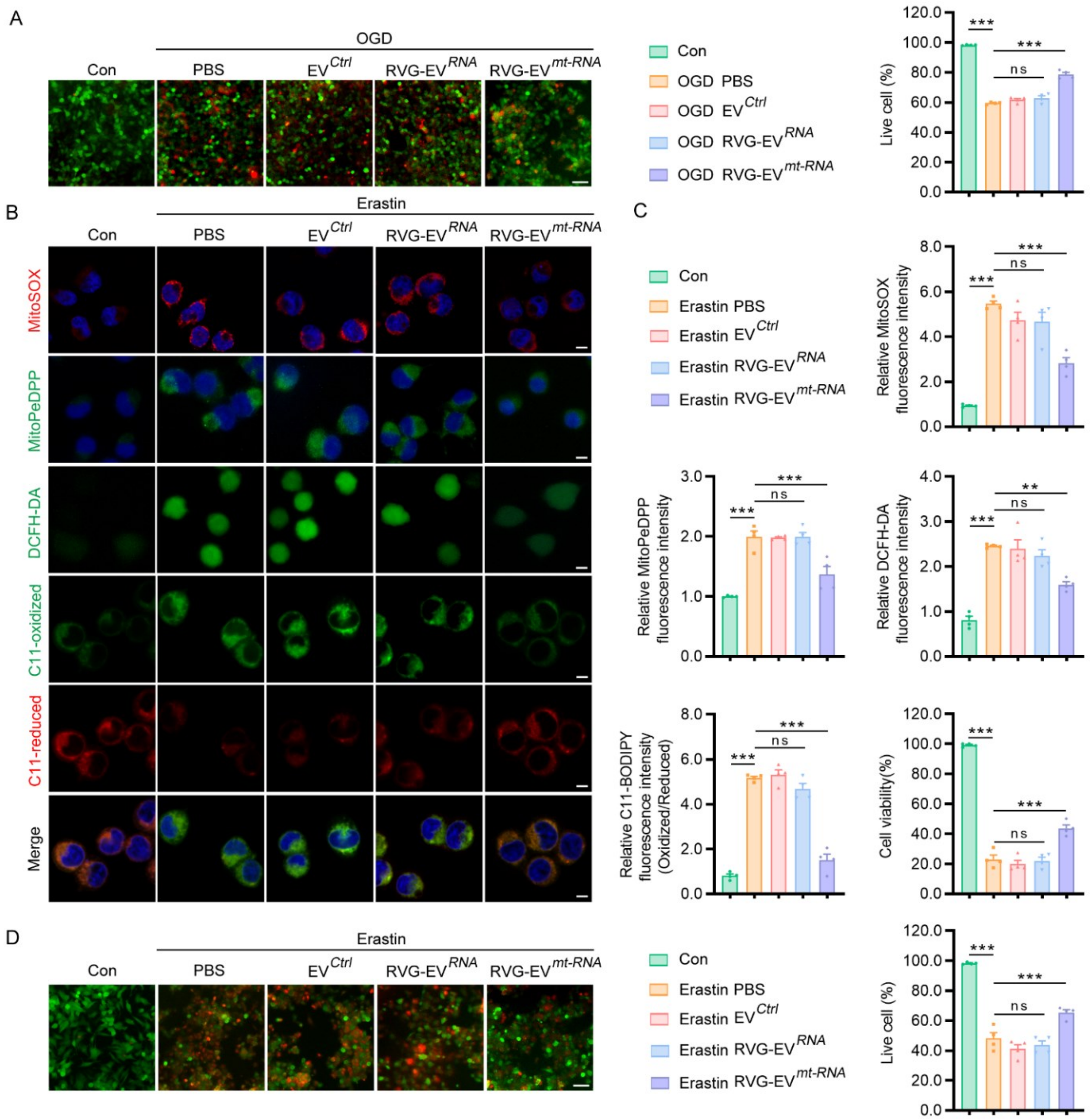

Figure S4. Extracellular vesicle-based circMTCO2 delivery attenuates erastin-induced ferroptosis in N2a cells.

(**A**) Representative fluorescence images and quantification of Live (green) /Dead (red) cell staining in N2a cells under control or OGD conditions, treated with PBS, EV^{Ctrl}, $RVG\text{-}EV^{RNA}$, and $RVG\text{-}EV^{mt\text{-}RNA}$. (**B**) Representative fluorescence images of MitoSOX, MitoPeDPP, DCFH-DA, and C11-BODIPY (oxidized and reduced forms) in control or erastin-treated N2a cells following PBS, EV^{Ctrl}, $RVG\text{-}EV^{RNA}$, or $RVG\text{-}EV^{mt\text{-}RNA}$ treatment. (**C**) Quantification of fluorescence intensities and cell viability. (**D**) Representative fluorescence images and quantification of Live (green) /Dead (red) cell staining. Scale bars, 50 μm (A, D), 5 μm (B). Data are presented as mean ± SEM (n = 4 per group). Statistical analysis was performed using an unpaired two-tailed Student's t-test and one-way ANOVA followed by Tukey's post hoc test. $^{**}P < 0.01$; $^{***}P < 0.001$; ns, not significant.

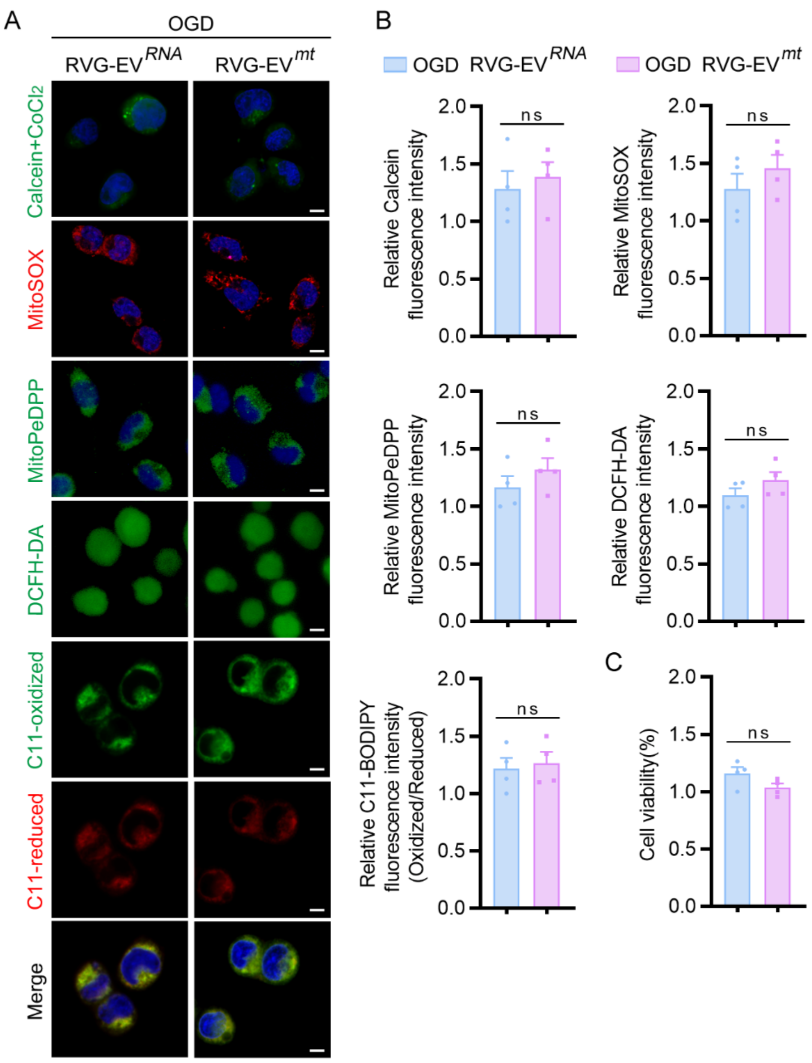

Figure S5. TPP-PDL alone does not confer mitochondrial protection under OGD conditions
(**A**) Representative fluorescence images of Calcein, MitoSOX, MitoPeDPP, DCFH-DA, and C11-BODIPY (oxidized and reduced forms) in N2a cells under OGD conditions, treated with RVG-EV^{RNA} or RVG-EV^{mt}. (**B, C**) Quantification of fluorescence intensities and cell viability assessed by Cell Counting Kit-8 (CCK-8) assay. Scale bars, 5 µm. Data are presented as mean ± SEM (n = 4 per group). Statistical analysis was performed using an unpaired two-tailed Student's t-test. ns, not significant.

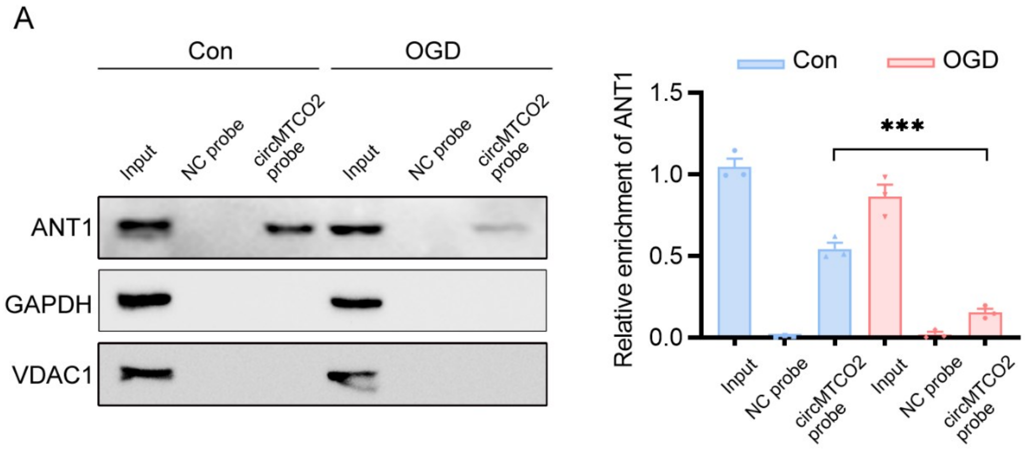

Figure S6. OGD disrupts circMTCO2-ANT1 interaction in N2a cells.

(**A**) Western blot of ANT1 pulled down by the biotinylated circMTCO2 probe under control (Con) and OGD conditions. GAPDH and VDAC1 served as cytoplasmic and mitochondrial controls, respectively. A biotin-labeled negative control (NC) probe was included to assess non-specific binding. Data are presented as mean ± SEM (n = 3 per group). Statistical analysis was performed using an unpaired two-tailed Student's t-test. ***$P < 0.001$.

A

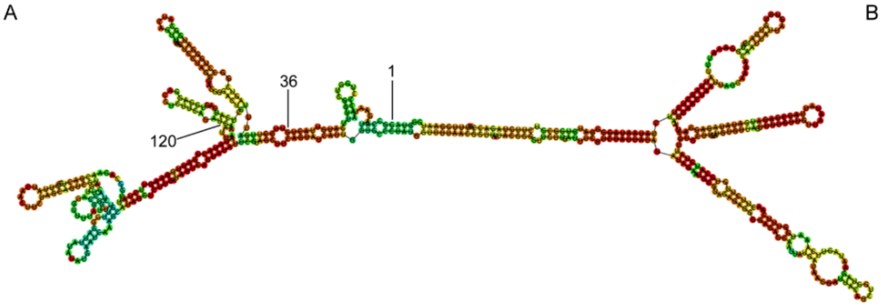

B

Input circMTCO2 fragment (nt)	Interaction propensity score
1-577	4.57
1-120	6.70
121-577	1.19
36-120	8.82

Figure S7. Prediction of circMTCO2 secondary structure and ANT1 interaction propensity. (**A**) RNAfold-predicted secondary structure of circMTCO2, with selected nucleotide positions indicated. (**B**) catRAPID-predicted interaction propensity scores between ANT1 and full-length or truncated circMTCO2 sequences. Higher scores indicate a greater predicted likelihood of interaction.

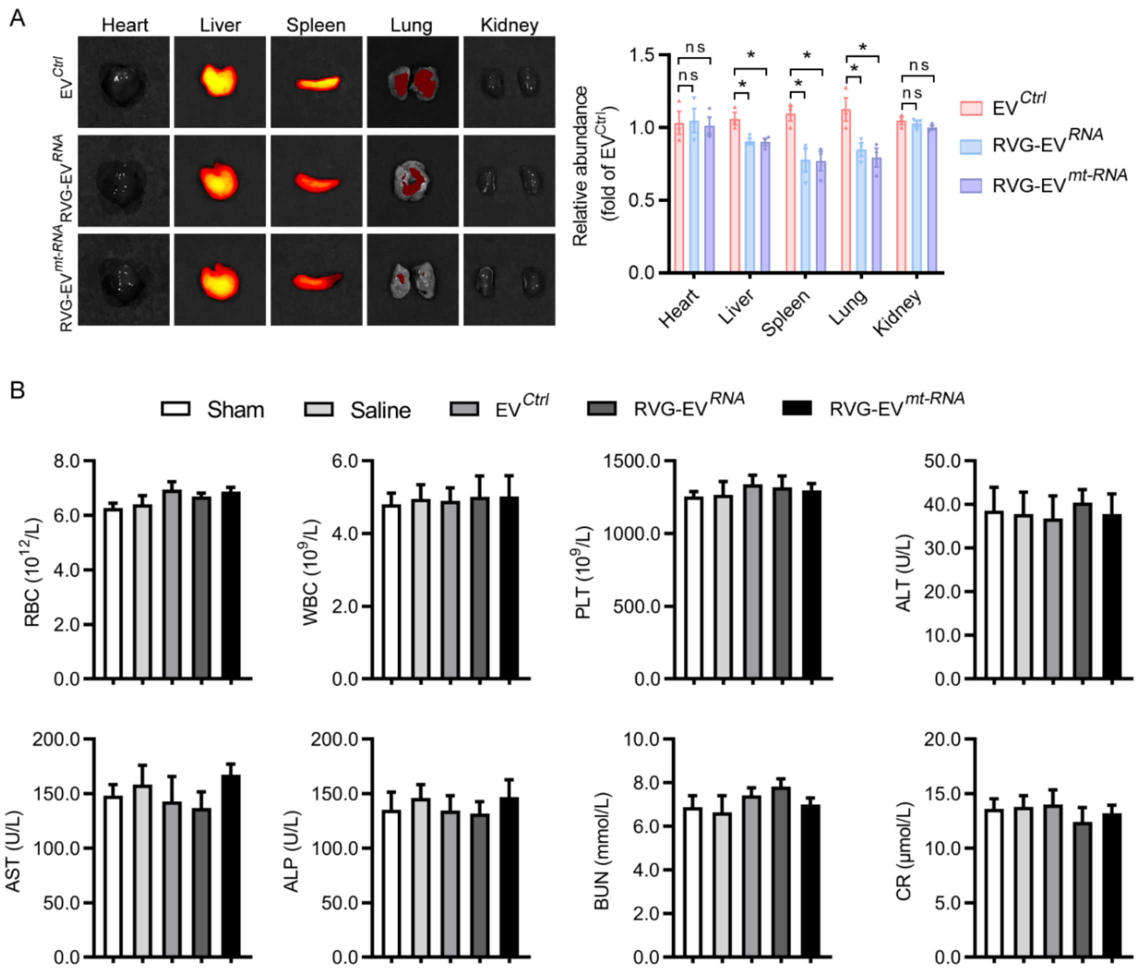

Figure S8. Major organ biodistribution and hematological/biochemical safety evaluation of RVG-EV$^{mt\text{-}RNA}$ therapy in vivo.

(**A**) Representative IVIS images and quantification of fluorescence signals in the heart, liver, spleen, lung, and kidney from mice treated with DiR-labeled EV^{Ctrl}, RVG-EV^{RNA} and RVG-EV$^{mt\text{-}RNA}$. For each organ, the signal was normalized to the corresponding EV^{Ctrl} group and presented as relative fluorescence signal (n = 3 per group). (**B**) Hematological (RBC, WBC, and PLT) and biochemical (ALT, AST, ALP, BUN, and CR) analyses of mice in the sham, saline, EV^{Ctrl}, RVG-EV^{RNA}, and RVG-EV$^{mt\text{-}RNA}$ groups at 7 dpi (n = 5 per group). Data are presented as mean ± SEM. Statistical analysis was performed using unpaired two-tailed Student's t-test and one-way ANOVA followed by Tukey's post hoc test. *$P < 0.05$; ns, not significant.

Supplementary Tables

Table S1. Primers and probes used in this study.

**qRT-PCR**	**Forward Primer**	**Reverse Primer**
circMTCO2 divergent primer	GGGGATGTGGCGTCTTGTAG	CAACAACCCCGTATTAACCGT
circMTCO2 convergent primer	AGCAGTCGTAGTTCACCAGG	GGGCACCAATGATACTGAAGC
linear MTCO2	CAGTCCCCTCCCTAGGACTT	CACAAATTTCAGAGCATTGGCC
Actb	GTGCTATGTTGCTCTAGACTTCG	ATGCCACAGGATTCCATACC
**probe**	**Sequence (5'-3')**	
circMTCO2 (for pulldown)	GTTATTCTATGGCCTACCCATTCCAACTTGG-biotin	
circMTCO2 (for FISH)	GTTATTCTATGGCCTACCCATTCCAACTTGG-digoxigenin	
circMTCO2 (1-40 nt)	GGCCATAGAATAACCCTGGTCGGTTTGATGTTACTGTTGC-biotin	
circMTCO2 (36-120 nt)	TTGCTTGATTTAGTCGGCCTGGGATGGCATCAGTTTTAAGTCCTAGGGAGGGGACTGCTCATGAGTGGAGGACGTCTTCAGATG-biotin	
circMTCO2 (121-260 nt)	AAATTAATATACGGATTGGAAGTTCTATTGGCAGAACGACTCGGTTATCAACTTCTAGCAGTCGTAGTTCACCAGGTTTTAGGTCGTTTGTTGGGATTATATATGAATCAAAGCATAGGTCTTCATAGTCAGTATATTCG-biotin	
circMTCO2 (261-420 nt)	TAGCTTCAGTATCATTGGTGCCCTATGGTTTTAACGGTTAATACGGGGTTGTTGATTTCGTCTATTATATATAGAATGCGTAGAGAGGGGAGAGCAATTATGATAAGGATTACAGCTGGTAGAATAGTTCAAATGGTTTCAACTTCTTGTGCATCTATTG-biotin	
circMTCO2 (421-532 nt)	TGCTTGTATGTGTTAGTTTTGTTGTTAATATTAGCGAGATGATATAGAGGACTAAGGAGCTAATTAGGAAAACAATTATTAGTGTGTGATCATGGAAATTTATTAGCTCTTCT-biotin	
circMTCO2 (533-577 nt)	ATAATAGGGGATGTGGCGTCTTGTAGACCAAGTTGGAATGGGTA-biotin	
negative control (NC) probe	AAAAAAAAAAAAAAAAAAAAAAAAA-biotin	

Table S2. List of antibodies used in this study.

Antibodies	Vendor	RRID
Rabbit anti-ANT1	ABclonal	AB_2763915
Rabbit anti-GAPDH	Cell Signaling Technology	AB_561053
Rabbit anti-VDAC1	ABclonal	AB_2862746
Rabbit anti-Lamp2b	Abcam	AB_2940865
Rabbit anti-CD63	Abcam	AB_2754982
Mouse anti-Tsg101	Abcam	AB_306450
Rabbit anti-GM130	Abcam	AB_880266
Normal rabbit IgG antibody	Cell Signaling Technology	AB_1031062
Anti-rabbit IgG, HRP-linked	Cell Signaling Technology	AB_2099233
Anti-mouse IgG, HRP-linked	Cell Signaling Technology	AB_330924
Sheep Anti-Digoxigenin Fab fragments	Roche	AB_514498
Guinea pig anti-NeuN	Millipore	AB_11205592
Mouse anti-4-Hydroxynonenal	R and D Systems	AB_664165
Mouse anti-Malondialdehyde	Thermo Fisher Scientific	AB_2735264
Goat anti-rabbit IgG secondary antibody Alexa Fluor 594	Thermo Fisher Scientific	AB_2534079
Goat anti-rabbit IgG secondary antibody Alexa Fluor 488	Thermo Fisher Scientific	AB_143165
